# A genome-scale metabolic network alignment method within a hypergraph-based framework using a rotational tensor-vector product

**DOI:** 10.1038/s41598-018-34692-1

**Published:** 2018-11-06

**Authors:** Tie Shen, Zhengdong Zhang, Zhen Chen, Dagang Gu, Shen Liang, Yang Xu, Ruiyuan Li, Yimin Wei, Zhijie Liu, Yin Yi, Xiaoyao Xie

**Affiliations:** 1 Key Laboratory of Information and Computing Science Guizhou Province, Guizhou Normal University, Guiyang, Guizhou China; 2 College of Mathematics and Information Science, Guiyang University, Guiyang, Guizhou China; 3 College of Mathematical Science, Guizhou Normal University, Guiyang, Guizhou China; 4 Key Laboratory of State Forestry Administration on Biodiversity Conservation in Karst of Southwest Areas China, Guizhou Normal University, Guiyang, Guizhou China; 5 School of Mathematics Sciences and Key Laboratory of Mathematics for Nonlinear Sciences, Fudan University, Shanghai, China

## Abstract

Biological network alignment aims to discover important similarities and differences and thus find a mapping between topological and/or functional components of different biological molecular networks. Then, the mapped components can be considered to correspond to both their places in the network topology and their biological attributes. Development and evolution of biological network alignment methods has been accelerated by the rapidly increasing availability of such biological networks, yielding a repertoire of tens of methods based upon graph theory. However, most biological processes, especially the metabolic reactions, are more sophisticated than simple pairwise interactions and contain three or more participating components. Such multi-lateral relations are not captured by graphs, and computational methods to overcome this limitation are currently lacking. This paper introduces hypergraphs and association hypergraphs to describe metabolic networks and their potential alignments, respectively. Within this framework, metabolic networks are aligned by identifying the maximal Z-eigenvalue of a symmetric tensor. A shifted higher-order power method was utilized to identify a solution. A rotational strategy has been introduced to accelerate the tensor-vector product by 250-fold on average and reduce the storage cost by up to 1,000-fold. The algorithm was implemented on a spark-based distributed computation cluster to significantly increase the convergence rate further by 50- to 80-fold. The parameters have been explored to understand their impact on alignment accuracy and speed. In particular, the influence of initial value selection on the stationary point has been simulated to ensure an accurate approximation of the global optimum. This framework was demonstrated by alignments among the genome-wide metabolic networks of *Escherichia coli* MG-1655 and *Halophilic archaeon* DL31. To our knowledge, this is the first genome-wide metabolic network alignment at both the metabolite level and the enzyme level. These results demonstrate that it can supply quite a few valuable insights into metabolic networks. First, this method can access the driving force of organic reactions through the chemical evolution of metabolic network. Second, this method can incorporate the chemical information of enzymes and structural changes of compounds to offer new way defining reaction class and module, such as those in KEGG. Third, as a vertex-focused treatment, this method can supply novel structural and functional annotation for ill-defined molecules. The related source code is available on request.

## Introduction

In recent years, whole-genome sequencing has been gradually completed for thousands of organisms, enabling a deeper and broader understanding of the functions represented by gene sequences^1^. Together with continuous improvements in determining the interactions among biological molecules, this sequencing effort has produced a huge number of biological networks at different scales for various species. Biological networks such as metabolic networks, protein-protein interaction networks and gene regulation networks can be used to describe the composition, status, and operation of biological systems^2,3^. Analysis of biological network, especially the genome-scale network, could systematically provide the collective patterns and common features of massive amounts of genome information can be studied as well as new biometric features and emergent phenomena^4^.

The flood of increasingly rich biological networks has accelerated the development and evolution of biological network alignment methods^5–7^. Biological network alignment aims to find a mapping between topological and/or functional components of different biological molecular networks. It can successfully address many essential biological questions, including the following^4,5,8^: which biological molecular interactions or groups of interactions are likely to have equivalent or conserved functions across species? In light of these similarities, can we predict novel functional information about components and interactions that are poorly characterized? Do these relationships inform us about the dynamics and evolution of molecules, networks and entire species?

Accordingly, numerous algorithms and tools have been developed for biological network alignment over the past decade^5,9–15^. For instance, Kelley and Sharan *et al*. matched two networks by searching high-ranking seeds in a dynamic programming method and extending around the seeds using a greedy strategy^5,9^. Pache *et al*. proposed a pairwise alignment approach with connected components as seeds^13^. Flannick *et al*. developed a multiple network aligner, Graemlin, which uses an incremental alignment approach by implementing successively pairwise alignments on the closest graph pairs^16^. Singh *et al*. used the idea of PageRank as the definition of similarities between vertices from different networks. And, they used a spectral graph method to rapidly identify the highest-ranking match from all possible matches in terms of the total score of all the aligned vertices^17^. Pržulj *et al*. exploited graphlet counts as topological node similarity scores and a greedy seed-and-extend method as the alignment strategy^11^. Heymans *et al*. performed metabolic network alignment by identifying a maximum weight matching of the enzyme similarity bipartite graphs. Pinter *et al*. converted the metabolic graph matching problem into a simple tree homeomorphism problem for alignment. Ay *et al*. proposed the SubMAP method, in which pathways are represented as compound-enzyme bipartite graphs and the alignment is converted into a conventional optimization problem^18^. Ay and his team again proposed a method that included a compression and decompression process of the pathways followed by the SubMAP. These efforts lead to a family of alignment methods that have swiftly evolved from a few early approaches into a repertoire of tens of methods^19,20^. The resulting methods have driven exploration and enhanced our understanding of the functional and organizational principles of different cellular processes.

These methods all rely on a graph representation. However, graph representation does not fully conform to reality of metabolic network, since metabolic reactions are obviously more complicated than can be described in simple graphs. A fundamental attribute of a graph is that each edge links two vertices. In contrast, metabolic reactions involve more than two participating components and are therefore not always bilateral^21^. Such multi-lateral relations cannot be captured by a graph.

Hypergraphs offer a framework to overcome such difficulties; biological networks can be intuitively described using the hypergraph model^21^. Klamt *et al*. and Mithani *et al*. proposed using a hypergraph to represent biological networks^21,22^. Michoel *et al*. have used hypergraph-based spectral clustering to perform protein-protein interaction networks classification^23^. Mohammadi *et al*. introduce a Triangular Alignment (TAME), which attempts to maximize the number of aligned triangles for 3-order protein-protein interaction network alignment based upon a tensor approach^24^.

This contribution introduces a hypergraph framework for metabolic network representation and develops a fast and easy alignment method through mathematical and computer improvements. Within this framework, metabolic networks are matched by identifying the maximal Z-eigenvalue of a symmetric tensor. A shifted symmetric higher-order power method was used to identify a solution that accurately approximates the global optimum. A rotational calculation strategy was designed to traverse all possible hyper-edges in Mohammadi’s implicit kernel of tensor-vector products for speed acceleration. The corresponding algorithm was realized with a Spark-based distributed memory computation on a cluster of 35 workers. These efforts attain a hundreds-fold increase in the convergence rate. Impact of certain parameters have been tested for this method. And, the influence of initial value on the convergent point has been investigated and a uniform vector has been found to be an appropriate choice. To demonstrate the framework, we apply it to aligning genome-scale metabolic networks of *Escherichia coli* MG1655 and *Halophilic archaeon* DL31.

This framework offers a completely intuitive, accurate, and comprehensive basis for the processing, management and analysis of metabolic networks. Because it is compatible with numerous tensor-based algorithms, this method will be benefit to a large family of downstream tools that could provide more in-depth insight into metabolic systems. The related source code is available on request.

## Results

### Illustration of a hypergraph-based metabolic network alignment

**Figure 1:**
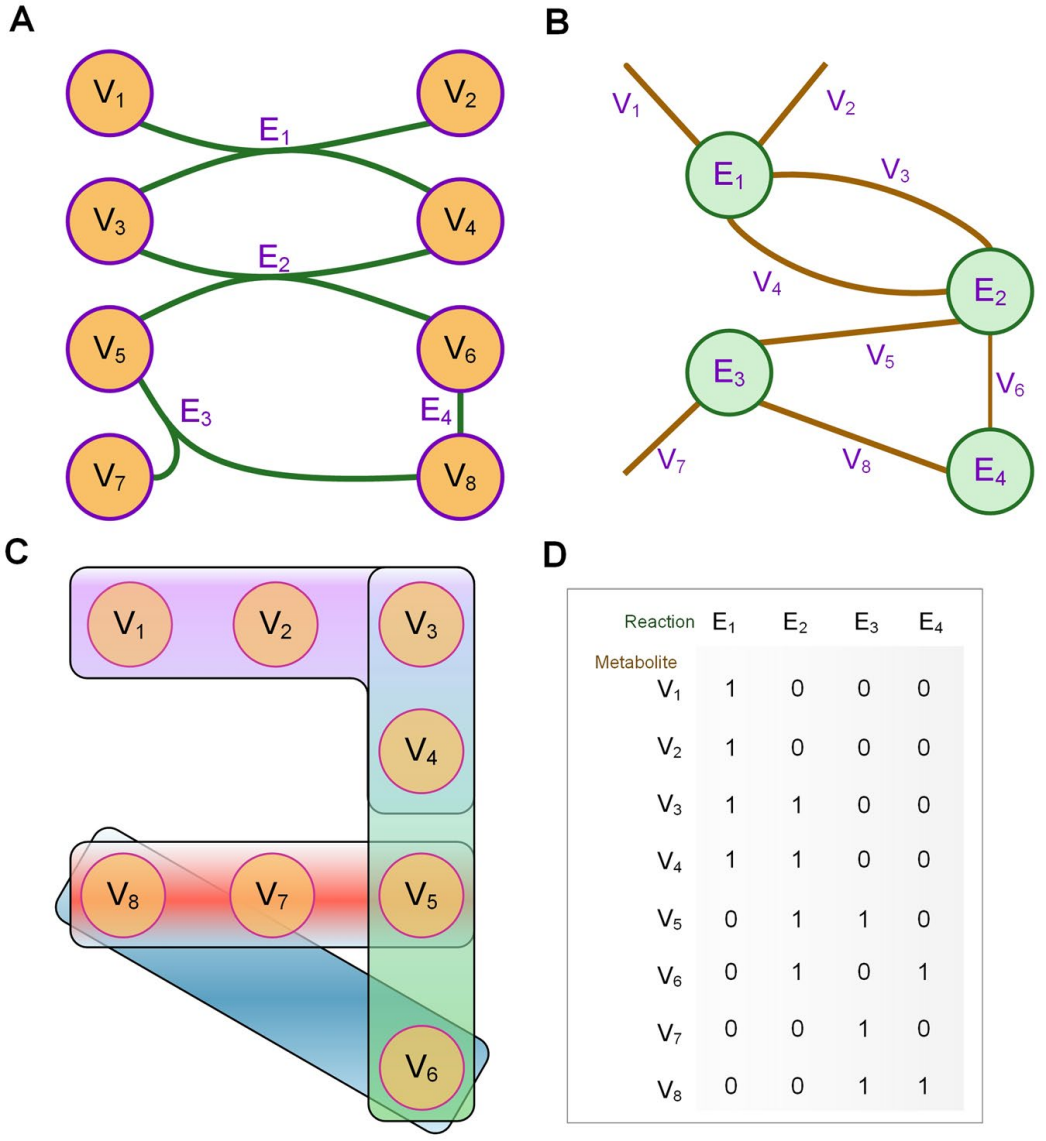
Different representation of metabolic network. (**A**) The original metabolic network. Circle of purple line and yellow fill represents the metabolites and green line represents the enzymatic reactions. (**B**) Simple graph representation of the metabolic network. Circle of green line and green fill represents the enzymatic reactions and brown line represents the metabolites. (**C**) Hypergraph representation of the metabolic network. Circle of purple line and yellow fill represents the metabolites and colorized blocks represent enzymatic reactions. E1: purple block, E2: green block, E3: red block. E4: blue block. (**D**) The hypergraph matrix of the metabolic network. Each column is representative of reaction while each row is representative of metabolite.

First, the difference of representing a metabolic network by a graph or by a hypergraph has been illustrated in Fig. 1. A metabolic network in Fig. 1A and its original storage format cannot be directly targeted by a simple graph.

Instead, it can only be represented by a reformatted simple graph using enzymes as the vertices and metabolites as the edges of the network, as shown in Fig. 1B. This reformat processing creates several problems. Substrates and metabolic end products become hovering edges connected to only one vertex. Parallel edges have been introduced between two vertices, leading to difficulties in map handling.

Fortunately, a hypergraph, in which edges can join more than two vertices (please see Method section), realize a more precise and comprehensive representation of metabolic network. This can be shown in Fig. 1C which is the corresponding hypergraph of Fig. 1A. And, Fig. 1D is the hypergraph matrix corresponding to the metabolic network.

**Figure 2:**
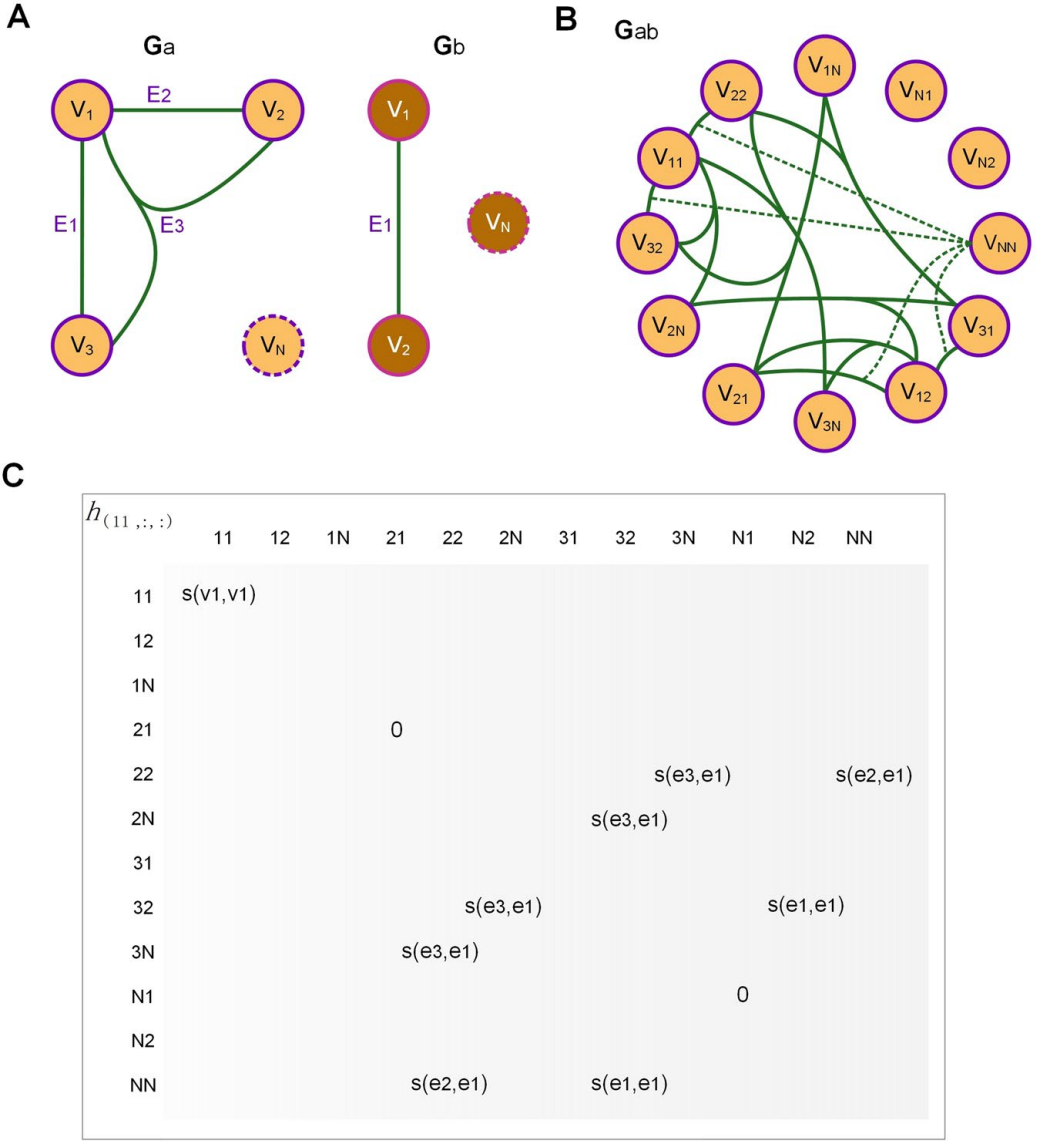
Illustration of hypergraph based metabolic network alignment. Circle of purple line and yellow fill and circle of purple line and brown fill represent the metabolites and green line represents the enzymatic reactions. (**A**) The two metabolic networks for alignment. Circles with dashed lines is null vertex N. Hypergraph a is of order 3 whereas hypergraph b is of order 2. (**B**) The associate hypergraph of the alignments. The subscript shows different alignments of the vertex in original network. V_NN_ represents the association vertex between the two null vertices. Dashed lines represent the association hyperedges containing V_NN_. This association hypergraph is of order 3. (**C**) The cross-section *h*(11, :, :) of the supersymmetric score tensor.

Figure 2 summarizes the metabolic network alignment using a toy hypergraph case. Network A contains 3 reactions and 3 compounds, whereas network B consists of only 1 reaction and 2 compounds (Fig. 2A). All of the potential alignments between edges and vertices are listed by generating an association hypergraph of the two hypergraphs *G*^*ab*^ (Fig. 2B). The association hypergraph can be generated so that its vertex corresponds to a potential pair of vertices of the two hypergraphs while its hyperedge a potential pair of hyperedges (please see Method section).

When conducting the alignment, we added a null vertex ‘*N’* to each hypergraph (metabolic network) to account for empty alignment or an absent compound, such as *V*_*1N*_. Therefore, the association hypergraph (Fig. 2B) contains 12 association vertices that represent the pairwise alignment of original vertices.

The number of association hyperedges will be more than association vertices. Alignments between any two enzymes can produce multiple association hyperedges, since the vertices subscript of one association hyperedges can be permutated to form other equivalent association hyperedges. For example, the alignment between *E*_2_ in *G*^*a*^ and *E*_1_ in *G*^*b*^ in Fig. 2A can produce two edges: *V*_*11*_*-V*_*22*_*-V*_*NN*_ and *V*_*12*_*-V*_*21*_*-V*_*NN*_. Here, when the dimension of association vertices is less than the tensor order, *V*_*NN*_ is used to fill the vacancies. The alignment between *E*_3_ and *E*_2_ can produce more hyperedge combinations.

As a graph represented by a matrix, the association hypergraph can be described by a tensor (please see Method section). A mathematical tensor is a multidimensional array of numerical values organized by their subscripts. For instance, a 2-dimentional tensor is a matrix. The element of such tensor is corresponding to the hypervertices and hyperedges of the association hypergraph. The value of each element is the similarity score of related association components. As such, association hypervertex is denoted by diagonal element and association hyperedge by non-diagonal element. This tensor is symmetric since non-diagonal elements’ value will not change when the subscripts are permutated. Figure 2C shows the cross-section *h*(11, :, :) of the super-symmetric score tensor of Fig. 2B.

### Tensor power iteration algorithm for hypergraph alignment

As described in the Methods section, the score of hypergraph alignment can be represented as a modal tensor-vector product^25,26^. Then, hypergraph alignment determines a vector *x* that maximizes the tensor multiplication according to certain constraints. The task for such a problem is seeking this vector and then discretizes it via a greedy algorithm^27^. This seeking task remains a NP-hard problem. Currently, we can only achieve an optimal approximate solution in the actual calculation^24,28^.

Several methods are available for this task. For example, maximizing the n-mode tensor product can be converted into a semi-definite programming problem via semi-definite relaxation, and a solution can be obtained via the primal-dual interior point method^29^. However, this method is computationally intensive and can only be used for networks with few vertices. Alternatively, tensor power iterations can be used to solve the problem^24,30,31^. This approach seeks the maximum Z-eigenvector and the eigenvalue of the tensor. Because the tensor is super-symmetric, this approach is equivalent to identifying the best symmetric rank-1 approximation of a symmetric tensor, and we adopt the shifted symmetric higher-order power method (SS-HOPM), which was introduced by Kolda *et al*.^30^. The corresponding procedure is provided in algorithm 1 (see Method section). The super-symmetric tensor *H* corresponds to the association hypergraph. The elements of *x* represent the alignment of vertices between two hypergraphs.

### Algorithm speed acceleration and storage reduction by rotational multiplication and distributed memory computing

Generally, genome-scale metabolic networks encompass hundreds to thousands of reactions, resulting in association hypergraphs of up to millions of hyperedges^32^. To ensure an accurate tensor-vector product, the hyperedge of the association hypergraph should be proliferated by permutating its subscript and filled into a symmetry tensor. A full representation of this symmetric tensor will introduce a K! fold increase in the number of variables. Such combinatorial explosion leads to massive storage need and, in particular, explosive computation requirement, which prevents the alignment on large networks.

**Figure 3:**
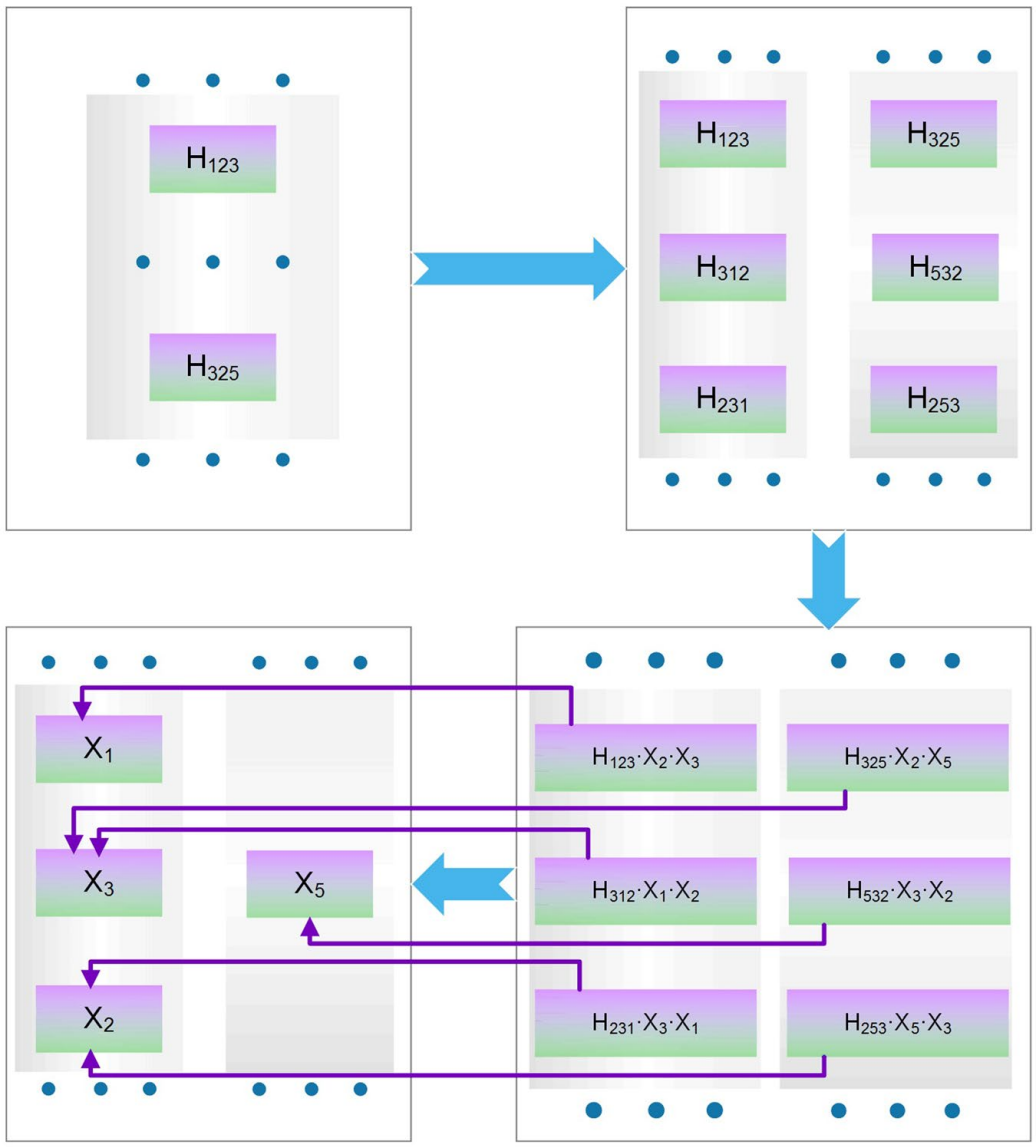
Illustration of rotational tensor-vector products algorithm. Symbol H in colorized block represents element in the HASH for the tensor. Symbol X in colorized block represents element in the HASH for the vector. Purple line means the contribution from different tensor elements to the ultimate vector element.

To this end, computational efforts have been realized to ensure the practicability of network alignment on a genome scale. The combinatorial explosion stemmed from the high-dimensional structure of tensor. After a careful study of the process of tensor-vector n-mode product, we found that this difficulty could be addressed by taking advantage of the symmetry of the tensor (Algorithm 2). We call this a rotational tensor-vector product for a super-symmetric tensor. In the process of production, the tensor could be fully represented by the elements with their size just as one upper hypertriangular region. Thus, it is only necessary to reconstruct such elements and store them in memory. An element in one position will contribute to elements on multiple positions of the resulting vector according to its subscript. To account for this feature, the elements are rotated according to its subscript to generate its K equivalents, each of which contribute to one position (Step 2 in algorithm 2). In addition, different elements contribute to the same position of the resulting vector as long as the last K-1 subscripts of these elements belong to the permutations of the same set of numbers. To this end, the new elements generated by rotation were multiplied by the permutation number of its last K-1 subscripts (Step 3 in algorithm 2). The flowchart of the entire process is displayed in Fig. 3.

We theoretically compared the computation and storage cost of the normal strategy and the rotational strategy. Given an association hypergraph with *|E*^*ab*^*|* edges, *|V*^*ab*^*|* vertices, K orders and its corresponding tensor. For a normal strategy, these features will result in an *|E*^*ab*^*|* * *K*! + *|V*^*ab*^*|* computation requirement and storage need. In comparison, for the rotational strategy the algorithm requires a computation cost of about *|E*^*ab*^*|* * *K* + *|V*^*ab*^*|* and the storage required is approximately *|E*^*ab*^*|* * *K*. Here, to trade space for time, we didn’t express the tensor for associate hypergraph as the Kronecker product of the tensors for original graph, which will achieve maximal storage cost saving as Mohammadi proposed.

**Figure 4:**
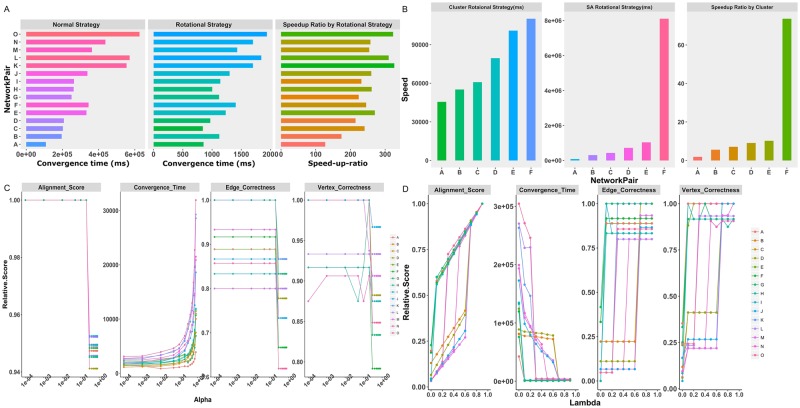
Speed and storage improvement by distributed computing and rotational algorithm and parameter impact on algorithm performance. (**A**) The speed-up ratio of rotational tensor-vector product strategy over normal one. The left figure is about convergent time run with normal strategy. Its x-axis represents convergent time at unit of millisecond. The middle figure is about convergent time run with rotational strategy. Its x-axis represents convergent time at unit of millisecond. The right figure is about speed-up ratio run of rotational strategy. Its x-axis represents the corresponding speed-up ratio of rotational strategy over normal one. Their y-axis represents 15 different network pairs. (**B**) The speed-up ratio of distributed rotational tensor-vector product method over stand-alone one. The left figure is about convergent time run with distributed rotational strategy. Its y-axis represents convergent time at unit of millisecond. The middle figure is about convergent time run with stand-alone rotational strategy. Its y-axis represents convergent time at unit of millisecond. The right figure is about speed-up ratio of distributed rotational strategy. Its y-axis represents the corresponding speed-up ratio of distributed rotational strategy over stand-alone one. Their x-axis represents 6 different network pairs. (**C**) Relationship between α and alignment speed and accuracy. The x-axis is α value and in logarithmic coordinates. The first figure is about the alignment score after discretization. Its y-axis represents relative alignment score after discretization normalized by the corresponding values at α = 0.0001. The second figure is about the convergence time. Its y-axis represents relative convergence time normalized by the corresponding values at α = 0.0001. The third figure is about the edge correctness. Its y-axis represents relative edge correctness normalized by the corresponding values at α = 0.0001. The fourth figure is about the vertex correctness. Its y-axis represents relative vertex correctness normalized by the corresponding values at α = 0.0001. (**D**) Relationship between λ and alignment speed and accuracy. The x-axis is λ value. The first figure is about the alignment score after discretization. Its y-axis represents relative alignment score after discretization normalized by the corresponding values at λ = 0.9. The second figure is about the convergence time. Its y-axis represents relative convergence time normalized by the corresponding values at λ = 0.9. The third figure is about the edge correctness. Its y-axis represents relative edge correctness normalized by the corresponding values at λ = 0.9. The fourth figure is about the vertex correctness. Its y-axis represents relative vertex correctness normalized by the corresponding values at λ = 0.9. The letters in legend of C and D represents 15 different network pairs. Please see Supplementary Data 2 for the network pairs used in these figures.

The algorithm incorporating rotational multiplication, which we call Rotational SS-HOPM (R-SS-HOPM), was first implemented in a stand-alone version. Further, distributed memory computing has been realized to ensure the feasibility of alignment for larger network, which we called Distributed SS-HOPM. The algorithm was implemented with Java APIs of the Spark framework on a cluster of up to 35 nodes (32G memory and 8 cores of 6700 K CPU). We compared their calculation speed with those of normal strategy on a stand-alone setting using selected network pairs. The speed-up ratio of the R-SS-HOPM over the normal one is reported in Fig. 4A (Please see Supplementary Data 1 and 2 for usage of the data set). The bar in the first figure is the convergence time of the normal SS-HOPM and that in the second figure is the convergence time of the R-SS-HOPM. The bar in the third figure is speed-up ratio of the R-SS-HOPM over the SS-HOPM. The R-SS-HOPM achieved an extraordinary acceleration. For the selected pairs, the R-SS-HOPM has an average speed-up ratio of 250 and a peak value over 300.

The speed-up ratio of the DR-SS-HOPM over R-SS-HOPM is reported in Fig. 4B. The bar in the first figure is the convergence time of DR-SS-HOPM and that in the second figure is the convergence time of R-SS-HOPM. The bar in the third figure is speed-up ratio of DR-SS-HOPM over R-SS-HOPM. From the results, DR-SS-HOPM, which takes advantages of distributed memory computing, has a further speed-up ratio of 15 in average over R-SS-HOPM and a peak value of 80. Such advantages will continue to increase as the mode of association hypergraph increases. So, R-SS-HOPM can satisfy the requirement of calculation at a genome-scale.

Since some metabolic reactions will encompass many metabolites, most genome-scale metabolic networks will have a large order. In aiding of the rotational multiplication strategy, the Distributed Rotational SS-HOPM becomes the most efficient and fast method to successfully treat such alignment tasks. We successfully aligned the complete metabolic networks of *Escherichia coli* (eco01100) and *Saccharomyces cerevisiae* (hah01100) which cannot be achieved by other methods in a short time.

### Impact of α and λ on alignment accuracy and convergence speed

A number of parameters can impact the performance of the algorithm. We tested their effects on the alignment speed and accuracy. Each network pair used for this purpose include one big network and one small network (details in Supplementary Data 1 and 2). The small network is a part of the big network, the network alignment is similar to self-alignment. This process is instructive given that correct vertex/edge matching is known before mapping and enables us to assess accuracy in a manner that is impossible when aligning hypergraphs with different sources. In these tests, alignment speed is measured by convergence time of the process. Alignment accuracy is quantified by various measures, including alignment score, edge correctness (EC) and vertex correctness (VC)^12^. Alignment score is the matching score of objective function in Eq. 4. EC indicates the percentage of edges from a smaller hypergraph that are correctly aligned to the edges in another hypergraph. VC similarly measures the fraction of nodes with correct alignment.

First, we tested the effects of the shift parameter α (please see Method section). An output alignment vector will be generated by iterative power multiplication on an input vector during each round of iteration. The algorithm will use both the output vector and the input vector to generate a vector as the new input vector. α represents the fraction of the old input vector retained in the new input vector. α is just like a sort of step size, less α means larger step size. The addition of the positive shift parameter α leads to a definite convexity of the objective function and ensures that the power method can converge^30^. In a preliminary investigation, we found that α near zero typically produced high-quality alignment, whereas α near one, which means nearly no optimization, caused large iteration steps and was not applicable to our algorithm. Thus, for each of the selected data, we changed α according to a list of (0.0001, 0.001, 0.01, 0.05, 0.1, 0.2, 0.3, 0.4, 0.5, 0.6, 0.7, 0.8) and recorded the convergence time and alignment accuracy with λ = 0.9 and the same random *x*_0_. The convergence time and accuracy for each α were normalized by the corresponding values at α = 0.1. Figure 4C presents the curve of α’s impact for each pair of networks. The alignment scores, ECs and VCs vary as α increases from 0 to approximately 1 and exhibits the same trend. A step response line shape is displayed and the highest value is achieved when α is less than 0.1. The convergence time exhibits a stable profile when α is between 0 and 0.05. When α becomes greater than 0.1, the convergence time increased dramatically for each network. This finding indicates that α generally increases both convergence speed and alignment accuracy. Why an increased in α impacts the alignment accuracy? Increased α means the convergence will be difficult to achieve. As such, in a real iteration, the numerical process will terminate at suboptimum. In the following calculation, α = 0.01 was used.

We then tested the impact of the balance factor λ on the convergence speed and alignment accuracies. The similarity score itself is dimensionless, so it is hard to weight the impact of vertices and edges in directing the optimization process (please see Method section). The factor λ has been introduced to adjust the weight of the two components. In final formula, hyperedge similarity was multiplied by (1 − λ) while hypervertex by λ. Higher λ lead to increasing weight of hypervertex in alignment score. So, this parameter will directly change the objective score and thus the direction of optimization process.

For each of the selected pairs, given *x*_0_ and α = 0.01, we recorded changes in the convergence speed and alignment accuracy for λ values in a list from 0 to 0.9 with a step of 0.1. The alignment score was normalized by the corresponding values at λ = 0.9 Fig. 4D shows that the alignment score increases significantly as λ increases. This occurs because the similarity scores of enzymes based on hierarchical taxonomy are significantly smaller than the CID similarity scores between compounds. Increased λ leads to decreased enzyme score fractions and increased metabolite score fractions, indicating a gradually increased total score.

For all the alignments, the curves of EC and VC versus λ looks two-valued and there is a threshold of λ for the curve. When λ is less than the threshold, EC and VC are relatively low. When λ becomes greater than the threshold, the alignment reaches relatively high EC and VC values. Meanwhile, increase of λ cause the drop of alignment speed of all networks. The reason for such phenomenon is because one pair of enzymes will give birth to a great deal of non-diagonal elements with equivalent values through the combination of the two sets of metabolites. This results in a large fraction of equivalent non-diagonal elements in the tensor, which greatly reduces the ability to identify correct alignments. Thus, it is difficult to converge to a high-quality alignment when λ is less than the threshold and non-diagonal elements exert a dominant influence on the alignment result. Conversely, this could explain why increased λ results in good alignment. This feature suggests that metabolite matching outperforms enzyme matching in determining the ultimate alignment result in current setting. In the following part, λ was set to 0.9.

In addition, many different score function can be used to align enzymes and metabolites^33,34^. Therefore, the value of λ is only a relative value and must be adjusted for different purposes.

### Impact on stationary point by initial vector selection

To a certain degree, the ultimate stationary points depend on initial values. To accurately approximate the global optimum, the impact of initial values on stationary points has been carefully explored with multiple rounds of parallel optimization using a random *x*_0_ as the initial point. The non-negativity of hypergraph tensor ensures the non-negativity of both the initial point and the optimum. In addition, a preliminary test shows that a uniform vector will always achieve an optimum with very high alignment accuracy. So, we generate the initial *x*_0_ by adding a non-negative random vector to a uniform vector *x*_*u*_. The fraction of the uniform component is controlled by coefficient β. In the following calculation, β ranges from 0 to 0.9 in a step of 0.1. For each β, 30 initial points were randomly generated as *x*_0_. For each network pair, the discretized score was normalized by the corresponding value based upon *x*_*u*_.

**Figure 5:**
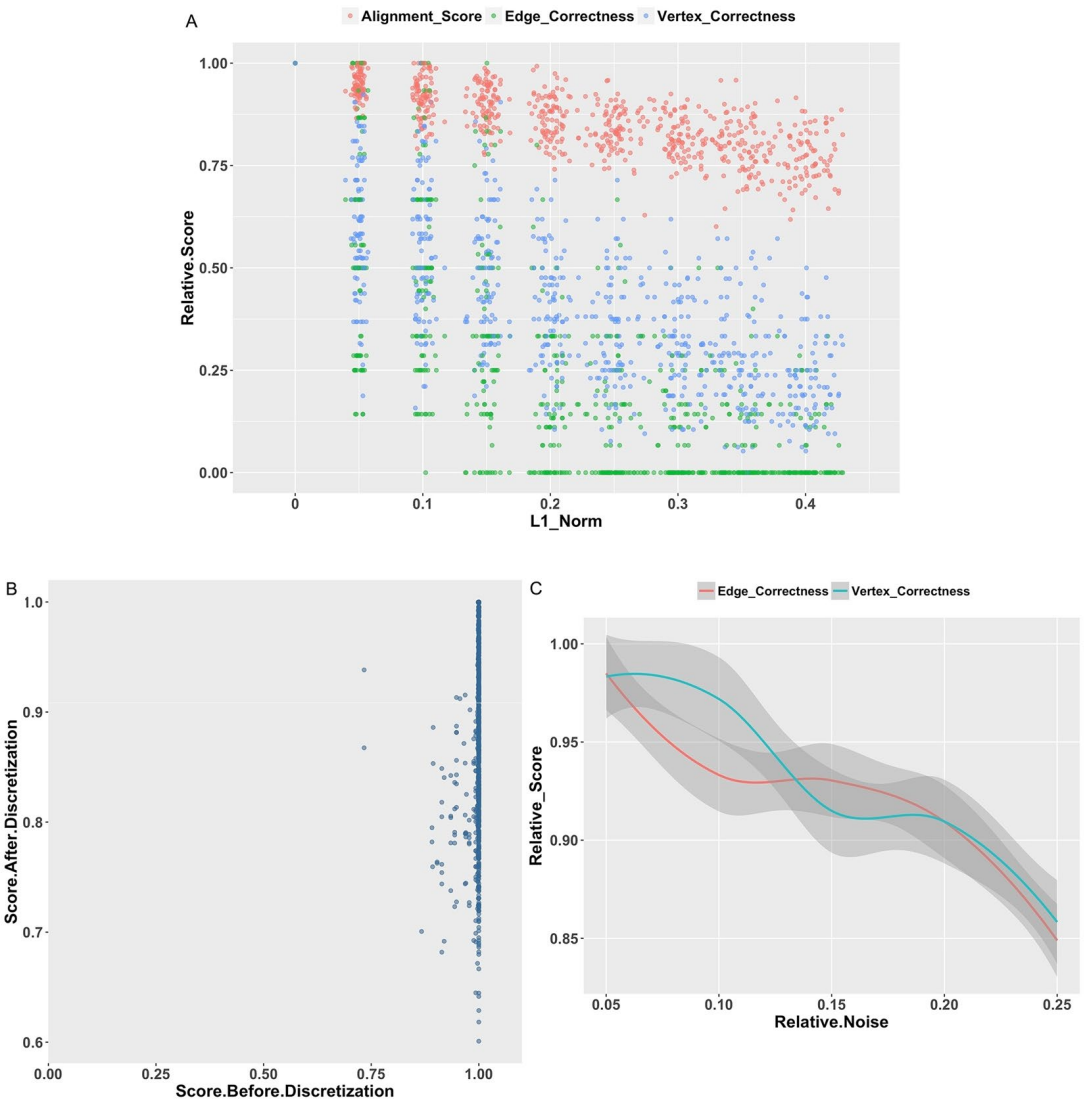
Property of the stationary points after convergence. The data for these figures are coming from 6 network pairs. (**A**) Relationship between initial vector selection and alignment score after discretization. X-axis shows the value of L1-norm of (*x*_0_–*x*_*u*_), which actually means the distance from initial vector to uniform vector. Y-axis shows the relative values of score after discretization, edge correctness and vertex correctness, which are normalized by the corresponding maximal scores for a same network pair. Red, green and blue dots represent the value of score after discretization, edge correctness and vertex correctness, separately. (**B**) Relationship between alignment score before discretization and alignment score after discretization. X-axis shows the relative value of score before discretization normalized by the corresponding maximal score for a same network pair. Y-axis shows the relative value of score after discretization normalized by the corresponding maximal score for a same network pair. (**C**) Performance of the algorithm on noisy network. Y-axis shows the relative values of edge correctness and vertex correctness normalized by the corresponding maximal scores for a same network pair. Red line is edge correctness and green line is vertex correctness from of average of different network pairs. Shaded gray area represents the error generated by a local polynomial regression fitting of the data by stat_smooth function of gglot2. Please see Supplementary Data 2 for the network pairs used in these figures.

Figure 5A shows the relationship between *x*_0_ and the distribution of alignment accuracy. Specifically, the x-axis represents values of the L1-norm of (*x*_0_–*x*_*u*_) ranging from 0 to 1, which actually represents the distance from *x*_0_ to *x*_*u*_, whereas the y-axis represents the alignment accuracy. Each dot is a stationary point of a single optimization process. The shape of the dots presents a downward trend. The alignment accuracy (regardless of whether EC, VC or alignment score is considered) always peaks when the L1-norm approaches zero. And, it decreases sharply as *x*_0_ moves away from *x*_*u*_. This finding suggests that optimization processes starting from *x*_*u*_ converge to the approximated maximum *x*_*m*_ in the space near *x*_*u*_ given that this stationary point is a satisfactory approximation of the global optimum in all feasible space. So, a uniform vector is the preferred initial point candidate.

We also studied the relationship between the objective function score and its discretized counterpart. This relationship is represented by the scatter plots in Fig. 5B. The horizontal and vertical coordinates of the scatter plot are all relative values normalized by the corresponding maximal score. Figure 5B shows that the vast majority of optimization with different initial points converges to stationary points with a very close score before discretization. However, although the scores before discretization are very close for each optimization, the scores after discretization and thus the alignment result differ significantly. The shapes of the dots indicate that the transformation from original score to discretized score exhibits a one-to-many relationship. This finding suggests that there are multiple maximums with similar importance spread across the space of a score before discretization, and few of these maximums map to the maximum in the space of a score after discretization. Therefore, a well-chosen initial point such as *x*_*u*_ is necessary to achieve a satisfactory approximation of the global optimum. For other experiments, *x*_*u*_ is chosen as the preferred initial point.

### The robustness of hypergraph-based method

We performed self-alignment with noise to assess the robustness of hypergraph-based method. The similarity score of each component in the association hypergraph was modified by simulated noise. The noise intensity was set at 5%, 10%, 15%, 20% and 25% of the mean intensity of the original alignment^35^. The true alignment is known because the networks are constructed using the same set of nodes.

The manner in which the alignments score varies as noise is shown in Fig. 5C. With the least noise, most alignments achieve a high matching quality. As the noise intensity increases, our method allows for a relatively slow decrease in matching quality. The performance suggests that it is robust to the presence of noise in the network.

### The alignment between the genome-scale metabolic network of Escherichia coli MG-1655 and Halophilic archaeon DL31

To assess the biological relevance produced by our method, we aligned the genome-scale metabolic networks of *Escherichia coli* MG-1655 (eco01100) and *Halophilic archaeon* DL31 (hah01100), both of which were obtained from KEGG PATHWAY module^36^.

**Figure 6:**
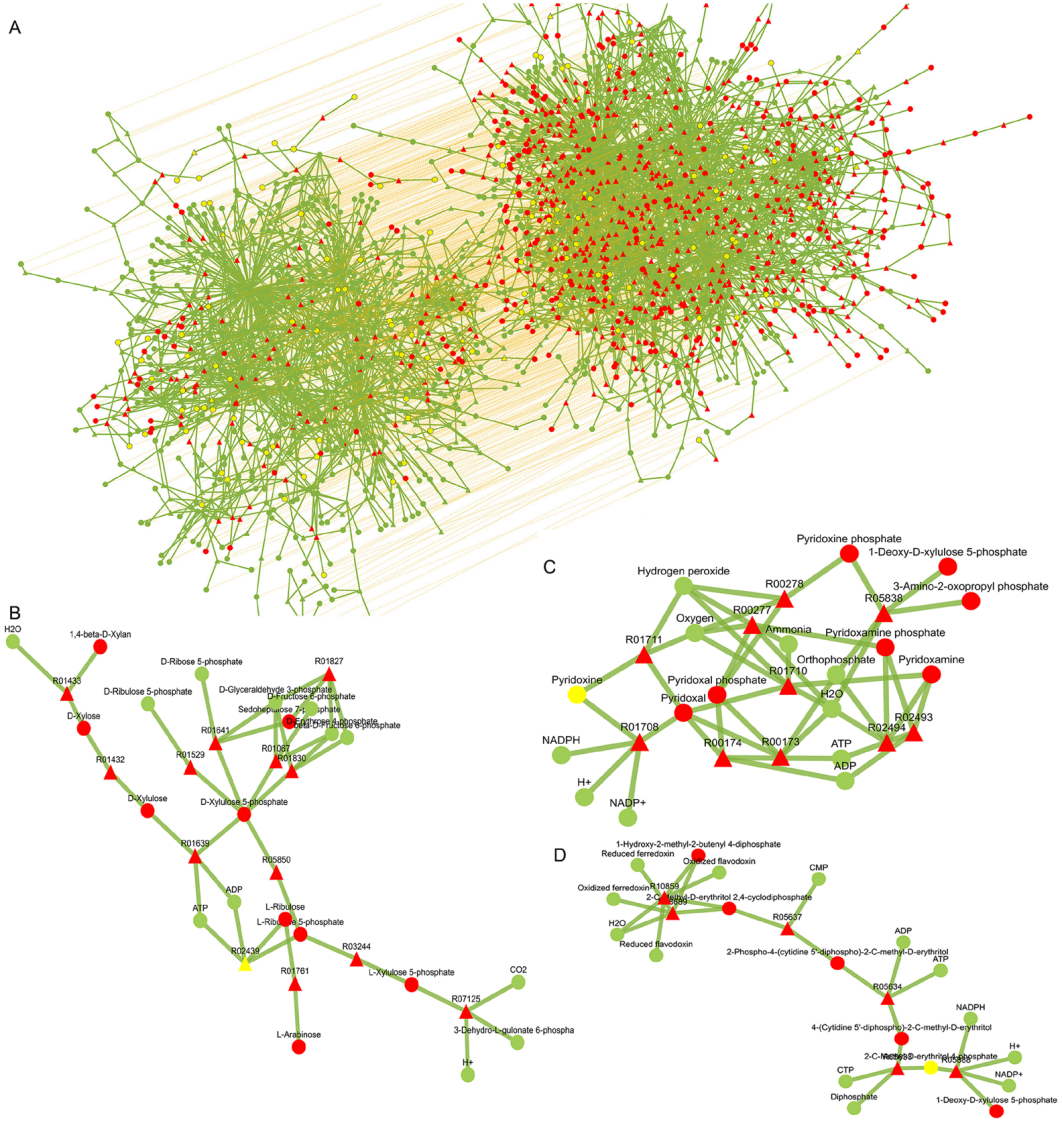
Alignment results for biosynthesis pathway of secondary metabolites between *Escherichia coli* MG-1655 and *Halophilic archaeon* DL31. The aligned networks are eco01110 and hah01110 from KEGG. Red dot represents unaligned metabolites, yellow dot represents missed aligned metabolites and green dot represents aligned metabolites. Red triangle represents unaligned reactions, yellow triangle represents missed aligned reactions and green triangle represents aligned reactions. The line represents the inclusion of metabolites in a reaction. (**A**) The whole alignment between eco01100 and hah01100. The curve represents the alignment relationship between the components. (**B**) An unaligned connected sub-network for non-oxidative pentose phospahte exchange and pentose and glucuronate interconversions in eco01100. R01067: D-Fructose 6-phosphate:D-glyceraldehyde-3-phosphate glycolaldehyde transferase. R01432: D -xylose aldose- ketose- isomerase. R01433: D-xylose xylohydrolase. R01529: D-Ribulose-5-phosphate 3-epimerase. R01639: ATP:D-xylulose 5-phosphotransferase. R01641: sedoheptulose-7-phosphate:D-glyceraldehyde-3-phosphate glycolaldehyde transferase. R01761: L-Arabinose aldose-ketose-isomerase. R01827: sedoheptulose-7-phosphate:D-glyceraldehyde-3-phosphate glyceronetransferase. R01830: beta-D-Fructose 6-phosphate:D-glyceraldehyde-3-phosphate glycolaldehyde transferase. R03244: L-ribulose- 5-phosphate 3 - epimerase. R02439: ATP: L- ribulose 5 phosphotransferase. R05850: L-ribulose-5-phosphate 4-epimerase. R07125: 3-dehydro-L-gulonate-6-phosphate carboxy-lyase. (**C**) An unaligned reaction combination in hah01100, which is related to MEP/DOXP pathway pathway. R00277: Pyridoxamine-5′-phosphate:oxygen oxidoreductase. R00278: Pyridoxine 5-phosphate:oxygen oxidoreductase. R01708: Pyridoxine:NADP+ 4-oxidoreductase. R01710: Pyridoxamine:oxygen oxidoreductase. R01711: pyridoxine:oxygen oxidoreductase. R00173: pyridoxal-5′-phosphate phosphohydrolase. R00174: ATP:pyridoxal 5′-phosphotransferase. R02493: ATP:pyridoxal 5′-phosphotransferase. R02494: pyridoxamine-5′-phosphate phosphohydrolase. R05838: pyridoxine 5′-phosphate synthase. (**D**) An unaligned connected sub-network in eco01100, which is related to methane metabolism. R05633: CTP: 2-C-Methyl-D-erythritol 4-phosphate cytidylyltransferase. R05634: ATP:4-(Cytidine 5′-diphospho)-2-C-methyl-D-erythritol 2-phosphotransferase. R05637: 2-Phospho-4-(cytidine 5′-diphospho)-2-C-methyl-D-erythritol CMP-lyase. R05688: 1-Deoxy-D-xylulose-5-phosphate isomeroreductase. R08689: (E)- 4-hydroxy- 3-methylbut -2-en-1-yl-diphosphate: oxidized ferredoxin oxidoreductase. R10859: (E)-4-hydroxy-3-methylbut-2-en-1-yl diphosphate:oxidized flavodoxin oxidoreductase.

The basic statistics of the alignment are listed in Table 1. The hah01100 network encompasses 537 reactions and 559 metabolites. Overall, 100% of the reactions were aligned, and 79.14% were accurately aligned. In addition, 100% of the metabolites were aligned, and 83.04% were accurately aligned. In comparison, the eco01100 network encompasses 923 reactions and 794 metabolites. Overall, 58.17% of the reactions were aligned and 46.05% were accurately aligned. In addition, 70.40% of the metabolites were aligned, and 58.56% were accurately aligned. A complete view of the alignments and their details are presented in Fig. 6A. Since most of the enzymes and metabolites aligned themselves correctly, we focused on the biological relevance of the missed match or gap.

**Table 1:** The basic statistics of the alignment between *Escherichia coli* MG-1655 and *Halophilic archaeon* DL31.

Network	Reaction Number	Metabolite Number	Match Ratio of Reaction	Accurate Match Ratio of Reaction	Match Ratio of Metabolite	Accurate Match Ratio of Metabolite
hah01100	537	559	1	0.7914	1	0.8304
eco01100	923	794	0.5817	0.4605	0.7040	0.5856

For eco0110 of *Escherichia coli* MG-1655, 498 enzymes and 329 metabolites were not aligned or correctly aligned to any of those in hah01100 of *Halophilic archaeon* DL31. A considerable proportion of the unaligned reactions and metabolites could be grouped together by their linkages, forming several separate pathways with clear biological function. There is no correct hit in hah0110 for enzymes including ATP:D-xylulose 5-phosphotransferase, D-xylose aldose-ketose-isomerase, sedoheptulose-7-phosphate:D-glyceraldehyde-3-phosphate glycolaldehyde transferase, D-xylose xylohydrolase, L-ribulose-5-phosphate 4-epimerase, L-ribulose-5-phosphate 3-epimerase, 3-dehydro-L-gulonate-6-phosphate carboxylyase, ATP:L-ribulose 5-phosphotransferase, beta-D-Fructose 6-phosphate:D-glyceraldehyde-3-phosphate glycolaldehyde transferase, sedoheptulose-7-phosphate:D-glyceraldehyde-3-phosphate glyceronetransferase, D-Ribulose-5-phosphate 3-epimerase and for metabolites including L-ribose, L-arabinose, D-xylose, D-xylulose, D-xylulose-5-phosphate, L-ribulose-5-phosphate, L-xylulose-5-phosphate. As shown in Fig. 6B, these enzymes and metabolites form parts of a sub-network responsible for non-oxidative pentose phospahte exchange and pentose and glucuronate interconversions, which mean that hah01100 do not possess such pathway. As such, for hah01110, some of the function of the pentose phosphate pathway was compensated for by its substitutes, such as the 2-deoxyribose 5-phophate aldolase (DERA) pathway and the 6-deoxy-5-ketofructose-1-phosphate (DKFP) pathway.

As shown in Fig. 6C, another such pathway is 2-C-methyl-D-erythritol-4-phosphate pathway or 1-deoxy-D-xylulose 5-phosphate pathway (MEP/DOXP pathway). This pathway is an alternative leading to the formation of isopentenyl pyrophosphate and dimethylallyl pyrophosphate. Thus, the alignment suggests that the hah01110 archaea possesses only the mevalonate pathway for its isoprenoid ether lipid production.

In addition, a number of cofactor pathways such as Vitamin B6, Vitamin B12 and biotin, as well as lipopolysaccharide biosynthesis pathway, have been found to be not fully aligned in eco01100 and thus to be partly missing in hah01100. These alignments show that hah01100 use modified versions of the pathways or it is inefficient in corresponding cofactors de novo synthesis and cell wall blocks producing, if hah01100 represents a complete annotation for the genome.

For hah01100 of *Halophilic archaeon* DL31, 112 enzymes and 94 metabolites were not aligned or correctly aligned to any of those in *Escherichia coli* MG-1655. There is no correct hit for enzymes of 5,10-Methenyl tetrahydro methanopterin 10-hydrolase, 5,10-Methylene tetrahydro methanopterin:coenzyme-F420 oxidoreductase and metabolites of 5,10-Methylene tetrahydro methanopterin, Reduced coenzyme F420, 5-Methyl-5,6,7,8-tetrahydro methanopterin, Coenzyme F420, 5,10-Methenyltetrahydromethanopterin, 5-Formyl-5,6,7,8-tetrahydro methanopterin in methane metabolism (in Fig. 6D). This indicates that the eco01110 is incompetent in methane biosynthesis and utilization and does not possess a full ability to grow on methane.

Most unaligned components are isolated and spread over the network and some of them belong to KEGG pathway of microbial metabolism in diverse environments (rn01120). It is not surprising in that as one extremophile, *Halophilic archaeon* DL31 inhabit extreme habitat with high salt stress and other distinctive nutrition requirement and environmental stress.

In a word, our alignment reveals the difference in nutrition requirement and metabolic capacity between eco01100 and hah01100 in details. Regardless of incomplete genome annotation, this will be a short reflection of their natural habitat and evolutionary history. Meanwhile, most of the results are also evidenced by rigorous experiments^37^, which in turn demonstrate the value of our method.

## Discussion

A hypergraph is a powerful tool with more profundity and applicability than a graph. Most complex relationships in the real world can be represented by hypergraphs. Therefore, it has increasing practical significance for extending the alignment of the metabolic network from a graph-based method to a hypergraph.

We adopted a power iteration method as the optimization strategy given its briefness and efficiency. Numerous modified versions have been generated during its evolution, such as the shifted power method or adaptive power method. In the power method without a shift, the iteration will always be rapid but will occasionally fail to converge. In contrast, sometimes the convergence rate is relatively slow for the adaptive method and the normal shifted power method with a large shift parameter. Thus, a compromise that satisfies both sides involves utilizing a small and fixed shifted parameter.

The greatest challenge of hypergraph alignment is the implementability of the entire process. Although the power method itself is relatively simple, it has not been successfully applied for large-scale and high-order biological networks such as metabolic networks in the real world. This limitation originates from the existence of too many elements in association with the hypergraph tensor. In constructing the association hypergraph, an alignment between any two hyperedges will produce association hyperedges with a number close to the factorial of the order. An alignment of two hyperedges includes both the alignment between two hyperedges and the alignment between the hypervertices belonging to each of the hyperedges. The number of possible alignments is close to a factorial of the order between the two sets of hypervertices.

Furthermore, since current eigen-pair enumeration methods are only accessible for super-symmetric tensors, we must permutate the subscripts of the association hyperedge to fill in the corresponding tensor. This process also leads to the explosive growth of the tensor elements. Consequently, the resultant tensor exhibits amazing memory consumption, and tensor-vector multiplication is extremely time-consuming for large-scale networks.

To this end, various efforts have been devoted to overcome these limitations. One effort involves implementing our algorithm within the architecture of distributed memory computing. This method possesses good scalability, and its ability can be enhanced with an increase in computing resources. It is suitable for the hypergraph with massive components, but not very high-order massive components. Compared with the stand-alone algorithm, the maximal speed-up ratio has reached the value of 80 by utilizing the full resources in this paper. However, for higher-order problems, the method will be difficult.

Another effort is to improve the algorithm performance using subscript rotational tensor-vector multiplication. This method is appropriate for speed acceleration and storage reduction of a high-order network. It could increase algorithm speeds by 250-fold while saving memory by tens of thousands-fold.

A joint method combining both of these efforts can achieve a speed-up ratio of 1000 and solve the problem that could not be addressed exclusively by either the stand-alone rotational method or distributed computing.

Several parameters have been examined to understand their influences on algorithm performance. α is one of the most dominant parameters. This parameter impacts the convexity of the objective function and then the convergence speed of the algorithm. α also affects the stationary point of the algorithm in our test. In practice, an α between 0.0001 and 0.01 is the preferred choice.

Another important parameter is λ. Strictly, λ is the parameter of the objective function instead of our algorithm. Its role is to balance the relative weight of the metabolites (hypervertex) over enzymes (hyperedge). In this paper’s test, λ exerts a great influence on the speed as well as alignment result. A larger λ indicates more weight of the metabolites, a faster convergence rate and a higher alignment score. The smaller the λ, the larger the weight of enzymes. Since enzymes (non-diagonal elements) have a very flat shape due to the equivalence of their values, the algorithm is not easy to reach a good stationary point when enzymes dominate the alignment. It is relatively difficult for an optimization with a small λ to converge. Although the weight of metabolites and enzymes is determined mainly by the specific structure and topology of the analyzed networks, the metabolites would have a stronger impact on alignment, from the formula of optimization algorithm itself. It is because that only if all the metabolites belonging to one enzyme (diagonal elements corresponding to one non-diagonal elements) correctly aligned, the algorithm wills generate an alignment of this enzyme. Additionally, the selection of similarity score function will also impact the alignment and interfere with λ. For instance, sequence homologous score of enzyme usually have a large value and will often let enzyme dominate the alignment. So, the influence of λ is complicated.

In essence, current power methods cannot guarantee the identification of global optima—they can only achieve a sufficient approximation of global optima. So, the challenge is how to ensure that the algorithm converges to such approximations. The choice of the initial vector itself has little influence on the alignment score, they give similar alignments with good approximation. However, our hypergraph alignment problem is subject to 0–1 constraints and must discretize the corresponding solution vector to obtain the ultimate alignment result. The discretized results are very sensitive to the stationary points before discretization. A little change in the alignment before discretization will lead to very different alignment result. In practice, the uniform vector is capable of converging to a very good approximation. Therefore, the uniform vector should be a preferred candidate for initial value selection.

Another possible method for achieving a satisfactory approximation of the global optimum might involve performing multiple rounds of parallel optimization using random *x*_0_. The alignment with the best score could be considered as an approximation of the global optimum. The number of samplings for achieving a global optimum may be associated with the size of the network itself. However, it is difficult to describe the relationship between the sampling number and the distance from the obtained optimal point to global optimum or the relationship between the sampling number and the probability to obtain the global optimum. The issue of how to determine an appropriate sampling number remains an open question.

As an example, an alignment among the metabolic networks of hah01100 and eco01100 was performed. The alignment difference was clarified in terms of enzymes and metabolites. Numerous significant biologically relevant differences were observed in non-oxidative pentose phospahte exchange and pentose and glucuronate interconversions pathway, MEX/DOXP pathway and some cofactor pathways. This numerical alignment has an excellent fit to the experimental results, which underscores the accuracy and power of this method.

## Conclusions

This study describes the background and a methodological framework for using hypergraphs to represent metabolic networks and tensors to align the networks. A hypergraph is suitable for intuitively, accurately and comprehensively describing metabolic networks. Association hypergraphs can be used to represent all possible alignments of the networks, whereas the corresponding tensor can store the similarity scores of the association hypergraph. This method provides an intuitive, accurate and comprehensive mathematical framework for the alignment of metabolic networks. A shifted symmetric higher-order power method was implemented on a spark-based computation cluster to solve the problem, significantly increasing the convergence rate up to 80-fold. A rotational tensor-vector product algorithm was introduced to accelerate the optimization by an average of 250-fold. The parameters have been simulated to determine their influence on alignment performance. In particular, the impact of the initial value on the convergence point has been tested to identify an accurate approximation of the global maximum. For the first time, this method achieved a genome-wide metabolic network alignment at both the metabolite level and the enzyme level. Similar to previous methods based upon a simple graph, a wide range of broad biological significance can be obtained using this hypergraph-based approach. In addition, it provides numerous valuable insights into bio-molecular networks. First, this method can access the driving force of chemical logic of organic reactions through the chemical evolution of metabolic network. Second, this method can incorporate the chemical information of enzymes and structural changes of compounds to offer new way defining reaction class and module, such as those in KEGG. Third, as a vertex-focused treatment, our method can supply novel structural and functional annotation for ill-defined molecules. Because this framework is compatible with numerous mathematical methods applicable to the tensor eigenvector problem, we believe it will form a set of tools with extensive applicability to metabolic network alignment and provide more in-depth insight into biological systems.

## Methods

### Association hypergraph representation of metabolic network alignment

A metabolic network containing m reactions and n metabolites can be accurately represented by hypergraph *G(V, E)* (although this study did not consider the direction of the reaction, the reaction direction can be naturally represented in a directed hypergraph). Generally, such a hypergraph *G* is a pair *G(V, E)* where *V* is a set of elements called vertices and *E* is a set of non-empty subsets of *V* called hyperedges. In this hypergraph, the set *E* = {*e*_*j*_, *j* = *1*, …, *m*} represents enzyme reactions in the metabolic network, whereas *V* = {*v*_*i*_, = *1*, …, *n*} represents metabolites in the metabolic network. The metabolic network can be represented by stoichiometric matrix *S*, in which a row corresponds to a metabolite and a column corresponds to a reaction. The element of *S* is the stoichiometric coefficient of a metabolite in a reaction with positive value standing for product and minus value standing for reactant. The stoichiometric matrix *S* can also be transformed into hypergraph matrix *G* after binarization of the stoichiometric coefficient. The order of hypergraph *K* is defined as the number of compounds of the reaction connected to the most compounds.

All of the possible alignment results between two hypergraphs *G*^*a*^ = (*V*^*a*^, *E*^*a*^) and *G*^*b*^ = (*V*^*b*^, *E*^*b*^) can be enumerated by constructing an association hypergraph *G*^*ab*^ of the two hypergraphs (Fig. 2A,B). The construction rule is that each vertex *V*^*ab*^ corresponds to a pair of vertices of the two hypergraphs while each hyperedge ${e}_{{a}_{1}{b}_{1},{a}_{2}{b}_{2},\cdots ,{a}_{K}{b}_{K}}$ corresponds to a pair of hyperedges in the two hypergraphs^38^. The order of the association hypergraph can be expressed as max {*K*_*a*_*, K*_*b*_}.

However, the alignment of the components becomes more complicated in the hypergraph. Since different enzymes may have different numbers of compounds, an alignment may occur between hyperedges with different orders (containing different numbers of compounds). This phenomenon necessitates the presentation of alignments between different numbers of vertices, such as the alignment between ${e}_{{a}_{1},{a}_{2},\cdots ,{a}_{m}}$ and ${e}_{{b}_{1},{b}_{2},\cdots ,{b}_{n}}$. To allow for the alignment of hyperedges with different orders, we introduce a null vertex, *N*, into each hypergraph. So, in the association hypergraph, vertices such as ${V}_{{a}_{i}N}$ and *V*_*NN*_ are generated to represent alignments including null vertices, which actually means components deletions or insertions in an alignment.

We then introduce the tensor *H* to represent the association hypergraph^39^. For simplicity, we define the tensor *H* that corresponds to the association hypergraph as super-symmetric because the similarity of a hyperedge does not depend on the order of the vertices of the hyperedge.

1 $$H:\{{h}_{{h}_{1},{h}_{2},\cdots ,{h}_{K}}\},\,{h}_{{h}_{1},{h}_{2},\cdots ,{h}_{K}}=S({e}_{{a}_{1},{a}_{2},\cdots ,{a}_{K}},\,{e}_{{b}_{1},{b}_{2},\cdots ,{b}_{K}})$$

The order of *H* is *K*, and the dimension of *H* is consistent with the vertices of the association hypergraph. The elements of *H*, ${h}_{{h}_{1},{h}_{2},\cdots ,{h}_{K}}$ represent the hyperedges of the association hypergraph. When the subscript of a tensor element indicates a half-empty vertex such as ${V}_{{h}_{i}N}$, it may represent an alignment between hyperedges with different numbers of vertices. When the subscript of a tensor element indicates a completely null vertex such as *V*_*NN*_ the orders of the two matching hyperedges are smaller than the order of the hypergraph. The function *S* acts as a degree of similarity measurement between the two hyperedges ${e}_{{a}_{1},{a}_{2},\cdots ,{a}_{K}}$ and ${e}_{{b}_{1},{b}_{2},\cdots ,{b}_{K}}$. Elements in which all subscripts of *h* are equal, i.e., elements on the tensor trace, represent the similarity measurements between the vertices of the two hypergraphs.

The values of the elements in the association hypergraph are the similarity measurements between the corresponding elements. For similarity among the vertices, we used a compound (metabolites) similarity score for which numerous metrics are available^40,41^. Specifically, we selected the similarity score calculated by ChemMine tools, which have an R interface to recognize the CID number of the compounds^42^. When a similarity score is missing for a compound pair, the value is set as an average of all compound pairs (please see Supplementary Data 3). For similarity between hyperedges, the specific values can be determined using hierarchical taxonomy or sequence similarity^33,34,43,44^. We used hierarchical taxonomy in this study. Briefly, we firstly determine the lowest class in the hierarchy shared by the EC number of the two enzymes. For example, considering Enzyme (1.1.1.1) and Enzyme (1.1.1.2), the lowest class is (1.1.1.-). Then, we calculate the similarity score as the inverse of the numbers of all enzymes belonging to this class.

We introduce the parameter λ ranging from 0 to 1 to balance the weights between the vertices and hyperedges^34^. The hyperedge similarity was multiplied by a factor of (1 − λ), whereas that of the hypervertex was λ. This parameter will directly affect the direction.

### Formalization of hypergraph matching

The alignment of two metabolic hypergraphs can be represented using the matrix *X* {*x*_*i*_ = 0, 1 | *x*_*i*_ ∈ *X*}^44^. The dimension of *X* is *n*^*a*^ * *n*^*b*^, which represents the vertices in the two hypergraphs. After the transformation of this matrix, we obtain a binary vector *X*. The elements of *X* are arranged in accordance with the sequence of the vertices of the two networks ${x}_{{a}_{i}{b}_{j}}=1$ represents the matching between the *i*th vertex of *G*^*a*^ and the *j*th vertex of *G*^*b*^. ${x}_{{a}_{i}{b}_{j}}=0$ represents no matching between the *i*th vertex of *G*^*a*^ and the *j*th vertex of *G*^*b*^. Then, the score of any alignment is a tensor product that can be represented by the following formula:

2 $$S(x)=H{\otimes }_{1}x{\otimes }_{2}x\,\cdots \,{\otimes }_{K}x$$

Thus, the hypergraph matching problem can be considered as a vector *x*^*m*^ that maximizes the tensor product under the above constraints, which is formalized as follows:

3 $$\begin{array}{c}{x}^{m}={\rm{\arg }}\,{\rm{\max }}\,S(x)\\ s.t.\,x\in {\{0,1\}}^{{n}^{a}{n}^{b}},\,\forall i\sum _{m=1}^{{n}^{a}}\,{x}_{{i}_{m}}\le 1,\,\forall m\sum _{i=1}^{{n}^{b}}\,{x}_{{i}_{m}}\le 1\end{array}$$

### Algorithm solving hypergraph matching

Currently, the solutions to the hypergraph matching problem of Eq. (3) all seek the optimal values of the equation first and then discretize these values via a greedy algorithm^27,34,45^. Despite relaxation, this problem remains a NP-hard problem, and we can only seek an optimal approximate solution in the actual calculation^27,31,45^. These approaches seek the maximum Z-eigenvector and the eigenvalue of this tensor. Given that the tensor is supersymmetric, this approach is equivalent to identifying the best symmetric rank-1 approximation of a symmetric tensor^46,47^, and the problem can then be solved using tensor power iterations based on spectral matching^48^. Here, we used the shifted symmetric higher-order power method (SS-HOPM), which was introduced by Kolda *et al*.^30^. Specifically, after the constraints of Eq. (3) are relaxed to 2-norm constraints, Eq. (4) can be obtained:

4 $$\begin{array}{c}{\rm{\max }}\,\gamma \\ s.t.\,H{\otimes }_{1}x{\otimes }_{2}x\,\cdots {\otimes }_{k-1}x={\rm{\gamma }}x,\,{x}^{T}x=1\end{array}$$

This algorithm sets an initial value that satisfies *x*^*T*^ *x* = 1 and then performs the following repeated iterative processes as Algorithm 1:

5 $${x^{\prime} }_{n+1}=H{\otimes }_{1}{x}_{n}{\otimes }_{2}{x}_{n}\,\cdots \,{\otimes }_{K-1}{x}_{n},\,{x}_{n+1}=\alpha {x}_{n}+(1-\alpha ){x^{\prime} }_{n+1}/{\Vert {x^{\prime} }_{n+1}\Vert }_{2}$$

where α is a shift parameter that affects the convergence speed and ranges from 0 to 1. This procedure converges to a stationary point, which is a good approximation of the global optimum.

Moreover, the stationary points obtained with this method depend on the initial value to a certain degree. The initial value is generated as the following:

6 $${x}_{0}=(1-\beta )\ast {x}_{r}+\beta \ast {x}_{u}$$

where *x*_0_ is the initial point, *x*_*r*_ is a random vector and *x*_*u*_ is a uniform vector.

In addition, the iterative result of this step is a continuous value. Therefore, discretization must be performed to convert the result into a binary permutation matrix, and we used the Kuhn-Munkres method for the discretization^49^.

### Implementation of the algorithm with reduced storage and accelerated speed

Considering the super-symmetry of the tensor, an efficient calculation strategy was designed to compute the tensor-vector product to achieve memory reduction and calculation acceleration. The scheme is displayed in Algorithm 2.

Because of the scarcity of the high-dimensional tensors, the data storage was organized into a HASH structure. A parallel version of the algorithm was implemented within the spark environment. The original HASH of the association hypergraph was split into small pieces and stored separately on different cores. The tensor-vector product has been calculated on this structure within each single core. Two key parts of the distributed algorithm are the representation of the higher-order tensor and the iterated multiplication of the tensor and vector. The tensor is packaged in JavaPair RDD, in which the key is the combination of the nonzero elements in the tensor and the value is the corresponding tensor value. In generating the RDD of Tensor, sc. parallelize was used to create the RDD of a possible combination of a compound and reaction pair. Then, mapPartitionsToPair was used for incremental encoding of key-values in the corresponding partition. Consequently, a repartition based on the partitionBy method was implemented by a remainder operation on the key code using the partition number as the module. Through this repartition operation, the distribution of key-values is roughly uniform among each partition. This optimization prevents data congestion on an individual worker, which typically leads to an OutOfMemoryError exception or slow computation. The algorithm ignored the intermediate low-order tensor to avoid data shuffling among nodes and reconstructed the ultimate vector with information from each node.

### Data set

The metabolic networks used in this study were obtained from the Kyoto Encyclopedia of Genes and Genomes (KEGG) pathway database using a KEGG API. Specifically, we obtained the pathways eco00010 (31 reactions), eco01110 (242 reactions) and eco01100 (923 reactions) of *Escherichia coli*, sce00010 (27 reactions) of *Saccharomyces cerevisiae*, hah01110 (168 reactions) and hah01100 (537 reactions) of *Halophilic archaeon*, and randomly knocked out certain reactions in the pathways. To construct randomly knockout pathways, we label the enzymes in the pathway with positive integer and randomly choose the integer, then delete the corresponding enzyme from the pathways. This gives birth to a series of different networks including eco00010-01 (21 reactions), eco00010-02 (15 reactions), eco00010-03 (9 reactions), eco00010-04 (6 reactions), sce00010-01 (21 reactions), sce00010-02 (15 reactions), sce00010-03 (12 reactions), hah01110-01 (100 reactions). Please see Supplementary Data 1 and 2 for more specific usage of the data set in the calculation^36^.

**Algorithm 1:**
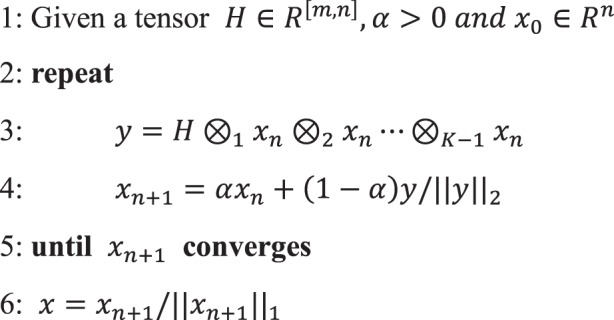
Higher-Order Power Method.

**Algorithm 2:**
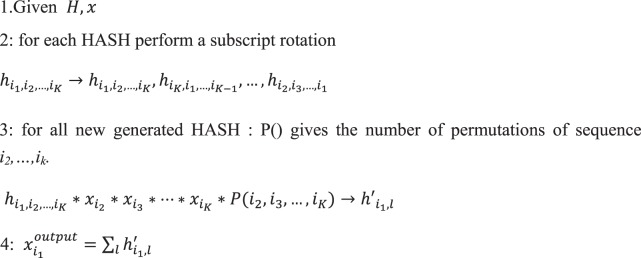
Rotary tensor-vector product for super symmetric tensor.

## Electronic supplementary material

Supplementary data 1

Supplementary data 2 and 4

Supplementary data 3

## Data Availability

All data generated or analysed during this study are included in this published article and its supplementary information files.
