# Supplementary material for: A genome-scale metabolic network alignment method within a hypergraph-based framework using a rotational tensor-vector product
Source: Sci Rep. 2018 Nov 6;8:16376. doi: 10.1038/s41598-018-34692-1 (PMC6219566; doi:10.1038/s41598-018-34692-1)
Supplement: Supplementary file 2 — Supplementary data 2 and 4 [file 41598_2018_34692_MOESM2_ESM.pdf]

# A genome-scale metabolic network alignment method within a hypergraph-based framework using a rotational tensor-vector product

Tie Shen[1+*], Zhengdong Zhang[2+], Zhen Chen[3+], Dagang Gu[2], Shen Liang[2], Yang Xu[1], Ruiyuan Li[1], Yimin Wei[5], Zhijie Liu[1], Yin Yi[4*], Xiaoyao Xie[1*]

[1]Key Laboratory of Information and Computing Science Guizhou Province, Guizhou Normal University, Guiyang, Guizhou, China

[2]College of Mathematics and Information Science, Guiyang University, Guiyang, Guizhou, China

[3]College of Mathematical Science, Guizhou Normal University, Guiyang, Guizhou, China

[4]Key Laboratory of State Forestry Administration on Biodiversity Conservation in Karst of Southwest Areas China, Guizhou Normal University, Guiyang, Guizhou, China

[5]School of Mathematics Sciences and Key Laboratory of Mathematics for Nonlinear Sciences, Fudan University, Shanghai, China

*Corresponding authors: xyxie@gznu.edu.cn, yyin@gznu.edu.cn and shentie@gznu.edu.cn

[+]These authors contributed equally to this work.

**Supplementary data 2:** The list of network pairs used for each figure.

1. The correspondence between letters in legend and network pairs in Fig. 4A is as following,
A: eco00010-03 vssce00010-03, B: eco00010-03 vs eco00010-02, C: eco00010-03 vs sce00010-02, D: eco00010-03 vs eco00010-01, E: eco00010-03 vs sce00010-01, F: sce00010-03 vs eco00010-02, G: sce00010-03 vs sce00010-02, H: sce00010-03 vs eco00010-01, I: sce00010-03 vs sce00010-01, J: eco00010-02 vs sce00010-02, K: eco00010-02 vs eco00010-01, L: eco00010-02 vs sce00010-01, M: sce00010-02 vs eco00010-01, N: sce00010-02 vs sce00010-01, O: eco00010-01 vs sce00010-01.
2. The correspondence between letters in legend and network pairs in Fig. 4C and 4D is as following,
A: eco00010 vs eco00010-02, B: eco00010-03 vs eco00010-02, C: eco00010-03 vs eco00010, D: eco00010-03 vs eco00010-01, E: eco00010 vs eco00010-01, F: eco00010-2 vs eco00010-04, G: eco00010-03 vs eco00010-04, H: eco00010-01 vs eco00010-04, I: sce00010-03 vs sce00010-01, J: sce00010-01 vs sce00010-02, K: sce00010-02 vs sce00010-03, L: sce00010-02 vs sce00010, M: sce00010-03 vs eco00010, N: sce00010 vs sce00010-01, O: sce00010-01 vs sce00010-01.

3. The correspondence between letters in legend and network pairs in Fig. 4B is as following,
A: hah01110-01 vs eco01110, B: hah01110-01 vs eco01110, C: hah01110-01 vs eco01110, D: hah01110-01 vs hah01110, E: hah01110-01 vs hah01110, F: hah01110-01 vs hah01110.
4. The data for Fig. 5 are coming from the following network pairs,
eco00010-03 vs sce00010-03, eco00010-03 vs eco00010-02, eco00010-03 vs sce00010-02, eco00010-03 vs eco00010-01, eco00010-03 vs sce00010-01, sce00010-03 vs eco00010-02, sce00010-03 vs sce00010-02, sce00010-03 vs eco00010-01, sce00010-03 vs sce00010-01, eco00010-02 vs sce00010-02, eco00010-02 vs eco00010-01, eco00010-02 vs sce00010-01, sce00010-02 vs eco00010-01, sce00010-02 vs sce00010-01, eco00010-01 vs sce00010-01, eco00010-02 vs eco00010-02, eco00010-02 vs eco00010-03, eco00010-02 vs eco00010-04, eco00010-03 vs eco00010-03, eco00010-03 vs eco00010-04 and eco00010-04 vs eco00010-04.

**Supplementary data 4:** Alignment result for hah01100 and eco01100 networks.

/root/big_networks/hah01100.csv reaction : 537, compounds : 559 -> /root/big_networks/eco01100.csv reaction : 923, compounds : 794
uniform x value##################################################
alpha : 0.01, lambda : 0.9, theta : 9
start powerMethodNormalize......
powerComputeXFactorial...
powerComputeXFactorial...
powerComputeXFactorial...
powerComputeXFactorial...
powerComputeXFactorial...
powerComputeXFactorial...
powerComputeXFactorial...
powerComputeXFactorial...
powerComputeXFactorial...
powerComputeXFactorial...
x iteration count : 10
score before discretize : 0.8999999999989009
discretize considering null node!
score after discretize : 479.62721299922094
compounds matched : total : 560, accurately matched : 465, accuracy : 83.04%
[3-Methylcrotonyl-CoA->Formyl-CoA, Thiocarboxy-[sulfur-carrier protein]->Thiocarboxy-[sulfur-carrier protein], Precursor Z->Precursor Z, beta-D-Fructose 1,6-bisphosphate->beta-D-Fructose 1,6-bisphosphate, Glycine->Glycine, Glutaryl-CoA->Glutaryl-CoA, N-Methylhydantoin->N-Methylhydantoin, N->UDP, N-(L-Arginino)succinate->N-(L-Arginino)succinate, N->CDP, 3-Oxooctanoyl-[acp]->3-Oxooctanoyl-[acp], Thiamin diphosphate->Thiamin diphosphate, D-Glucono-1,5-lactone 6-phosphate->D-Glucono-1,5-lactone 6-phosphate, S-Adenosyl-L-homocysteine->S-Adenosyl-L-homocysteine, dUMP->dUMP, N->Urate, N->5-Hydroxyisourate, 3-Methyl-2-oxobutanoic acid->3-Methyl-2-oxobutanoic acid, dAMP->dAMP, N->5-Hydroxy-2-oxo-4-ureido-2,5-dihydro-1H-imidazole-5-carboxylate, 3-Oxododecanoyl-CoA->3-Oxododecanoyl-CoA, N->(S)-Allantoin, N->Allantoate, alpha-D-Glucosamine 1-phosphate->alpha-D-Glucosamine 1-phosphate, 3-Hydroxyglutaryl-[acp] methyl ester->3-Hydroxyglutaryl-[acp] methyl ester, 5-Formyl-5,6,7,8-tetrahydromethanopterin->UDP-N-acetylmuramoyl-L-alanyl-gamma-D-glutamyl-L-lysine, 15-cis-Phytoene->di-trans,poly-cis-Undecaprenyl phosphate, N->Butanoic acid, Adenosine->Adenosine, N->Xanthine, DNA cytosine->DNA cytosine, (R)-3-Ureidoisobutyrate->(R)-3-Ureidoisobutyrate, Xanthosine->Xanthosine, ADP->ADP, 3'-Phosphoadenylyl sulfate->3'-Phosphoadenylyl sulfate, L-Asparagine->L-Asparagine, 3-Sulfinylpyruvate->3-Sulfinylpyruvate, 2,5-Diamino-6-(5-phospho-D-ribitylamino)pyrimidin-4(3H)-

one->5-Amino-6-(5'-phospho-D-ribitylamino)uracil, UDP->N, (R)-3-Amino-2-methylpropanoate->(S)-3-Hydroxyisobutyrate, N->(S)-Lactate, N->Ferricytochrome c, N->Ferrocytochrome c, Protein asparagine->Dimethylbenzimidazole, N->Acetyl phosphate, N->Hypoxanthine, Fe2+->Fe2+, Isochorismate->Isochorismate, L-2-Aminoadipate->N, [Enzyme]-cysteine->[Enzyme]-cysteine, L-Adrenaline->N, N->Enzyme N6-(dihydrolipoyl)lysine, tRNA(Glu)->tRNA(Glu), N->[Dihydrolipoyllysine-residue succinyltransferase] S-glutaryldihydrolipoyllysine, (8S)-3',8-Cyclo-7,8-dihydroguanosine 5'-triphosphate->(8S)-3',8-Cyclo-7,8-dihydroguanosine 5'-triphosphate, (3R)-3-Hydroxytetradecanoyl-[acyl-carrier protein]->(3R)-3-Hydroxytetradecanoyl-[acyl-carrier protein], N->Enzyme N6-(lipoyl)lysine, dTMP->dTMP, 5,10-Methenyltetrahydromethanopterin->UDP-3-O-(3-hydroxytetradecanoyl)-D-glucosamine, Acetaldehyde->Acetaldehyde, (S)-Malate->(S)-Malate, trans-Dec-2-enoyl-CoA->trans-Dec-2-enoyl-CoA, L-Citrulline->L-Citrulline, Serotonin->N, Hydrochloric acid->N2-Succinyl-L-arginine, Thiamin triphosphate->Thiamin triphosphate, meso-2,6-Diaminoheptanedioate->meso-2,6-Diaminoheptanedioate, N->S-Adenosylmethioninamine, (3R)-3-Hydroxyoctanoyl-[acyl-carrier protein]->(3R)-3-Hydroxyoctanoyl-[acyl-carrier protein], Phosphoenolpyruvate->Phosphoenolpyruvate, L-Homocysteine->L-Homocysteine, 3-Ketoglutaryl-[acp] methyl ester->3-Ketoglutaryl-[acp] methyl ester, Adenosyl cobyrinate a,c diamide->N, CO2->CO2, DNA 5-methylcytosine->DNA 5-methylcytosine, PQQ->PQQ, UDP-N-acetyl-alpha-D-glucosamine->UDP-N-acetyl-alpha-D-glucosamine, 3-Hydroxypimeloyl-[acp] methyl ester->3-Hydroxypimeloyl-[acp] methyl ester, N->S-Ribosyl-L-homocysteine, (R)-5,6-Dihydrothymine->(R)-5,6-Dihydrothymine, Oxaloacetate->Oxaloacetate, D-Ribulose 5-phosphate->D-Ribulose 5-phosphate, Adenosine 3',5'-bisphosphate->Adenosine 3',5'-bisphosphate, N->O-Succinyl-L-homoserine, N->Cytosine, alpha-Aminoadipate carrier protein LysW->N, N->D-Ribose, CDP->N, 3-Sulfino-L-alanine->3-Sulfino-L-alanine, 2,5-Diamino-6-(5-phospho-D-ribosylamino)pyrimidin-4(3H)-one->2,5-Diamino-6-(5-phospho-D-ribosylamino)pyrimidin-4(3H)-one, trans-Hex-2-enoyl-CoA->trans-Hex-2-enoyl-CoA, L-Glutamate->L-Glutamate, GMP->GMP, 3-Oxohexadecanoyl-[acp]->3-Oxohexadecanoyl-[acp], IMP->IMP, DNA->DNA, Nicotinamide D-ribonucleotide->Nicotinamide D-ribonucleotide, Thiosulfate->Thiosulfate, 3-Oxooctanoyl-CoA->3-Oxooctanoyl-CoA, O-Phospho-L-homoserine->O-Phospho-L-homoserine, L-Aspartate->L-Aspartate, D-Erythrose 4-phosphate->D-Erythrose 4-phosphate, 5'-Phosphoribosylglycinamide->5'-Phosphoribosylglycinamide, 2-Methylbut-2-enoyl-CoA->2-Methylbut-2-enoyl-CoA, S-Methyl-5-thio-D-ribose 1-phosphate->beta-D-Glucose 1-phosphate, UDP-glucose->UDP-glucose, Glutaminyl-tRNA->N, Ferricytochrome cL->Ferricytochrome cL, 3-(Imidazol-4-yl)-2-oxopropyl phosphate->3-(Imidazol-4-yl)-2-oxopropyl phosphate, Thiamine->Thiamine, L-erythro-4-Hydroxyglutamate->L-erythro-4-Hydroxyglutamate, 3-Oxodecanoyl-[acp]->3-Oxodecanoyl-[acp], Putrescine->Putrescine, Diphosphate->Diphosphate, L-Tyrosine->4-Methylthio-2-oxobutanoic acid, Tetradecanoyl-CoA->Tetradecanoyl-CoA, Thymidine->Thymidine, Polyphosphate->Polyphosphate, Acetyl-CoA->Acetyl-

CoA, Ethanol->Ethanol, all-trans-Octaprenyl diphosphate->all-trans-Octaprenyl diphosphate, Formaldehyde->N, UMP->UMP, Tetrahydropteroyltri-L-glutamate->Tetrahydropteroyltri-L-glutamate, N-(5'-Phospho-D-1'-ribulosylformimino)-5-amino-1-(5''-phospho-D-ribosyl)-4-imidazolecarboxamide->N-(5'-Phospho-D-1'-ribulosylformimino)-5-amino-1-(5''-phospho-D-ribosyl)-4-imidazolecarboxamide, Lipoylprotein->Lipoylprotein, 5-Methyltetrahydrofolate->5-Methyltetrahydrofolate, N->Hydroxyproline, (S)-2-Aminobutanoate->(S)-2-Aminobutanoate, Phosphatidylglycerophosphate->Phosphatidylglycerophosphate, L-Tryptophan->L-Tryptophan, 3-Ketopimeloyl-[acp] methyl ester->3-Ketopimeloyl-[acp] methyl ester, L-Threonine->L-Threonine, GDP-mannose->GDP-mannose, UTP->N, Ferrocytochrome c->N, dCTP->dCTP, Decanoyl-CoA->Decanoyl-CoA, (R)-Pantoate->(R)-Pantoate, 3-Oxotetradecanoyl-CoA->3-Oxotetradecanoyl-CoA, 1-(5'-Phosphoribosyl)-5-formamido-4-imidazolecarboxamide->1-(5'-Phosphoribosyl)-5-formamido-4-imidazolecarboxamide, D-Alanine->D-Alanine, 2-Demethylmenaquinone->2-Demethylmenaquinone, (4R)-4-Hydroxy-2-oxoglutarate->(4R)-4-Hydroxy-2-oxoglutarate, Sulfite->Sulfite, Ferrocytochrome cL->Ferrocytochrome cL, Agmatine->Agmatine, Palmitoyl-CoA->Palmitoyl-CoA, 5-Hydroxyindoleacetate->2-Maleylacetate, 5-Phosphoribosylamine->5-Phosphoribosylamine, 1-(5'-Phosphoribosyl)-5-amino-4-imidazolecarboxamide->1-(5'-Phosphoribosyl)-5-amino-4-imidazolecarboxamide, N4-Acetylaminobutanal->N, sn-Glycerol 3-phosphate->sn-Glycerol 3-phosphate, D-erythro-1-(Imidazol-4-yl)glycerol 3-phosphate->D-erythro-1-(Imidazol-4-yl)glycerol 3-phosphate, 3-Dehydro-L-gulonate 6-phosphate->3-Dehydro-L-gulonate 6-phosphate, Citrate->Citrate, Indole->Indole, trans-Hexadec-2-enoyl-CoA->trans-Hexadec-2-enoyl-CoA, (S)-2-Methylbutanoyl-CoA->(S)-3-Hydroxyisobutyryl-CoA, D-Ribose 5-phosphate->D-Ribose 5-phosphate, alpha-D-Glucose->alpha-D-Glucose, Dihydrolipoylprotein->Dihydrolipoylprotein, L-Methionine->L-Methionine, Urea->Urea, 2-Dehydro-3-deoxy-6-phospho-D-gluconate->2-Dehydro-3-deoxy-6-phospho-D-gluconate, 2-Succinyl-5-enolpyruvyl-6-hydroxy-3-cyclohexene-1-carboxylate->2-Succinyl-5-enolpyruvyl-6-hydroxy-3-cyclohexene-1-carboxylate, L-Serine->L-Serine, RNA->RNA, Nucleoside triphosphate->Nucleoside triphosphate, L-Arginine->L-Arginine, Chloroacetic acid->Propanoate, Acetate->Acetate, 2-trans-Dodecenoyl-CoA->2-trans-Dodecenoyl-CoA, 10-Formyltetrahydrofolate->10-Formyltetrahydrofolate, D-Glyceraldehyde->D-Glyceraldehyde, Ferricytochrome c->N, Glycerol->Glycerol, FMN->FMN, L-Histidinal->L-Histidinal, LysW-gamma-L-lysine->2-Oxo-3-hydroxy-4-phosphobutanoate, D-Aspartate->D-Aspartate, L-Alanine->L-Alanine, Glutathione->Glutathione, Folate->Folate, Lauroyl-CoA->Lauroyl-CoA, 3-Methylbutanoyl-CoA->3-Hydroxypropionyl-CoA, Coenzyme F420->UDP-N-acetylmuramoyl-L-alanine, 4-Methyl-2-oxopentanoate->4-Methyl-2-oxopentanoate, L-Kynurenine->L-Arogenate, cis-3-Chloroallyl aldehyde->cis-3-Chloroallyl aldehyde, (3R)-3-Hydroxypalmitoyl-[acyl-carrier protein]->(3R)-3-Hydroxypalmitoyl-[acyl-carrier protein], Mercaptopyruvate->Mercaptopyruvate, Cardiolipin->Cardiolipin, 3-Phospho-D-glyceroyl phosphate->3-Phospho-D-glyceroyl

phosphate, S-Aminomethyldihydrolipoylprotein->S-Aminomethyldihydrolipoylprotein, Protoporphyrin->Protoporphyrin, Lipoyl-[acp]->Lipoyl-[acp], LL-2,6-Diaminoheptanedioate->LL-2,6-Diaminoheptanedioate, dGDP->dGDP, Oxalosuccinate->Oxalosuccinate, LysW-gamma-L-alpha-aminoadipyl 6-phosphate->N, L-Phenylalanine->L-Phenylalanine, 2-Oxobutanoate->2-Oxobutanoate, Oxidized ferredoxin->Oxidized ferredoxin, trans-Tetradec-2-enoyl-CoA->trans-Tetradec-2-enoyl-CoA, Phylloquinone->Phylloquinone, 3-Oxo-OPC4-CoA->3-Oxo-OPC4-CoA, 5-Amino-6-(1-D-ribitylamino)uracil->5-Amino-6-(1-D-ribitylamino)uracil, 5'-Phosphoribosyl-N-formylglycinamide->5'-Phosphoribosyl-N-formylglycinamide, 1,4-Dihydroxy-2-naphthoyl-CoA->1,4-Dihydroxy-2-naphthoyl-CoA, dGMP->dGMP, Methylamine->N, L-2,4-Diaminobutanoate->L-Glutamate 5-semialdehyde, G00008->Nicotinate D-ribonucleoside, 1D-myo-Inositol 3-phosphate->1D-myo-Inositol 3-phosphate, D-Gluconic acid->D-Mannonate, Ammonia->Ammonia, 7,8-Dihydroneopterin 3'-triphosphate->7,8-Dihydroneopterin 3'-triphosphate, N->N, N->2-Amino-3-oxo-4-phosphonooxybutyrate, N->3-Amino-2-oxopropyl phosphate, N->Thymine, Heme O->Heme O, N->2-Hydroxy-3-oxopropanoate, L-Histidinol phosphate->L-Histidinol phosphate, cis-3-Chloro-2-propene-1-ol->cis-3-Chloro-2-propene-1-ol, FADH2->FADH2, 4-Amino-2-methyl-5-(phosphooxymethyl)pyrimidine->4-Amino-2-methyl-5-(phosphooxymethyl)pyrimidine, Cys-Gly->Cys-Gly, D-Glucose->D-Glucose, N->N-Methylputrescine, N->1-Methylpyrrolinium, UDP-glucuronate->UDP-glucuronate, N->Cadaverine, L-Leucine->L-Leucine, 3,4-Dihydroxyphenylacetaldehyde->3,4-Dihydroxymandelaldehyde, N->5-Aminopentanal, Acetoacetyl-CoA->Acetoacetyl-CoA, Dolichyl phosphate->N1-(5-Phospho-alpha-D-ribosyl)-5,6-dimethylbenzimidazole, dGTP->dGTP, D-Mannose 1-phosphate->D-Mannose 1-phosphate, S-Adenosyl-L-methionine->S-Adenosyl-L-methionine, Phenylpyruvate->Phenylpyruvate, N-Carbamoylsarcosine->N, Squalene->N, Deoxyguanosine->Deoxyguanosine, N-Carbamoyl-L-aspartate->N-Carbamoyl-L-aspartate, Glutamate->Glutamate, N->4-Hydroxybenzoate, N->tRNA, N->tRNA containing 6-isopentenyladenosine, L-Lysine->L-Lysine, L-1-Pyrroline-3-hydroxy-5-carboxylate->L-1-Pyrroline-3-hydroxy-5-carboxylate, 2-(Formamido)-N1-(5'-phosphoribosyl)acetamidine->2-(Formamido)-N1-(5'-phosphoribosyl)acetamidine, 5-Methyl-5,6,7,8-tetrahydromethanopterin->UDPMurNAc(oyl-L-Ala-D-gamma-Glu-L-Lys-D-Ala-D-Ala), 3-Oxopalmitoyl-CoA->3-Oxopalmitoyl-CoA, 1,4-Dihydroxy-2-naphthoate->1,4-Dihydroxy-2-naphthoate, Prephytoene diphosphate->(S)-Hydroxydecanoyl-CoA, 7-Cyano-7-carbaguanine->7-Cyano-7-carbaguanine, D-Glyceraldehyde 3-phosphate->D-Glyceraldehyde 3-phosphate, Protoporphyrinogen IX->D-Urobilinogen, Hexadecanoic acid->Hexadecanoic acid, 3-Methoxy-4-hydroxyphenylacetaldehyde->N, Isopenicillin N->N, 4-Aminobenzoate->4-Aminobenzoate, (S)-3-Hydroxybutanoyl-CoA->(S)-3-Hydroxybutanoyl-CoA, N->4-Hydroxy-3-polyprenylbenzoate, N->2-Polyprenylphenol, N->2-Polyprenyl-6-hydroxyphenol, cis-3-Chloroacrylic acid->Aminoacrylate, N->2-Polyprenyl-6-methoxyphenol, N->Phosphatidate, N->1-Acyl-sn-glycerol 3-phosphate, N->2-Polyprenyl-3-methyl-5-hydroxy-6-methoxy-1,4-benzoquinone, N->n D-Glucose,

N->1,2-Diacyl-sn-glycerol, N-Acetylputrescine->N-Acetylputrescine, Geranyl diphosphate->Geranyl diphosphate, 3-Oxotetradecanoyl-[acp]->3-Oxotetradecanoyl-[acp], UDP-N-acetyl-D-mannosamine->UDP-N-acetyl-D-mannosamine, L-Selenocysteine->L-Selenocysteine, N->D-Gluconic acid, N->D-Glutamate, N->2-Keto-D-gluconic acid, (R)-5-Phosphomevalonate->2-C-Methyl-D-erythritol 4-phosphate, N->Ubiquinone, N->Selenophosphoric acid, N->Ubiquinol, N->Hypotaurine, N->Creatinine, Succinate->Succinate, L-Ornithine->L-Ornithine, N->Spermine, N->L-Cysteate, N->Taurine, Hydroquinone->Hydroquinone, Adenosyl cobyrinate hexaamide->N, 2-Methylacetoacetyl-CoA->2-Methylacetoacetyl-CoA, IDP->IDP, 2-Succinylbenzoate->2-Succinylbenzoate, Aminoimidazole ribotide->Aminoimidazole ribotide, Dimethylallyl diphosphate->Dimethylallyl diphosphate, L-3,4-Dihydroxybutan-2-one 4-phosphate->L-3,4-Dihydroxybutan-2-one 4-phosphate, Phytyl diphosphate->Phytyl diphosphate, N->tRNA(Gln), N->gamma-L-Glutamyl-L-cysteine, N->Glutaminyl-tRNA, Deoxyadenosine->Deoxyadenosine, N->D-Glutamine, myo-Inositol 4-phosphate->myo-Inositol 4-phosphate, 5,10-Methylenetetrahydrofolate->5,10-Methylenetetrahydrofolate, Reduced coenzyme F420->UDP-N-acetylmuramoyl-L-alanyl-D-glutamate, dTTP->dTTP, Guanosine->Guanosine, Adenylyl-[sulfur-carrier protein]->Adenylyl-[sulfur-carrier protein], 3-Dehydroquinate->3-Dehydroquinate, 7-Carboxy-7-carbaguanine->7-Carboxy-7-carbaguanine, L-Proline->L-Proline, 3-Phospho-D-glycerate->3-Phospho-D-glycerate, CDP-diacylglycerol->CDP-diacylglycerol, N->Spermidine, Prephenate->Prephenate, N->Glutathionylspermidine, N->5-Methylthio-D-ribose, L-Cystathionine->L-Cystathionine, N->Oxalyl-CoA, Reduced acceptor->Reduced acceptor, 3-Oxohexanoyl-CoA->3-Oxohexanoyl-CoA, H+->H+, FAD->FAD, 2-Amino-4-hydroxy-6-hydroxymethyl-7,8-dihydropteridine->2-Amino-4-hydroxy-6-hydroxymethyl-7,8-dihydropteridine, 4-Aminobutanoate->4-Aminobutanoate, Sulfur donor->Protein N6-(octanoyl)lysine, Penicillin->N, 6,7-Dimethyl-8-(D-ribityl)lumazine->6,7-Dimethyl-8-(D-ribityl)lumazine, N->Di[3-deoxy-D-manno-octulosonyl]-lipid IV(A), Dihydropteroate->Dihydropteroate, N->3-Deoxy-D-manno-octulosonate, N->3-Deoxy-D-manno-octulosonate 8-phosphate, N->D-Arabinose 5-phosphate, N->Lauroyl-KDO2-lipid IV(A), N->Di[3-deoxy-D-manno-octulosonyl]-lipid A, N->ADP-D-glycero-beta-D-manno-heptose, N->D-glycero-beta-D-manno-Heptose 1-phosphate, N->D-glycero-beta-D-manno-Heptose 1,7-bisphosphate, Menaquinone->Menaquinone, (R)-Lipoate->(R)-Lipoate, NADP+->NADP+, Adenine->Adenine, Reduced flavodoxin->Reduced flavodoxin, 5,10-Methylenetetrahydromethanopterin->UDP-3-O-(3-hydroxytetradecanoyl)-N-acetylglucosamine, N->(Z)-3-Ureidoacrylate peracid, N->(Z)-3-Peroxyaminoacrylate, N->UDP-2,3-bis(3-hydroxytetradecanoyl)glucosamine, N->Carbamate, N->3-Hydroxypropanoate, N->Lipid A disaccharide, N->alpha,alpha'-Trehalose 6-phosphate, N->2,3,2'3'-Tetrakis(3-hydroxytetradecanoyl)-D-glucosaminyl-1,6-beta-D-glucosamine 1,4'-bisphosphate, N->alpha,alpha-Trehalose, N->3-Deoxy-D-manno-octulosonyl-lipid IV(A), N->CMP-3-deoxy-D-manno-octulosonate, N->2-Dehydro-3-deoxy-D-arabino-heptonate 7-phosphate, L-Glutamine->L-Glutamine, 2-

Succinylbenzoyl-CoA->2-Succinylbenzoyl-CoA, 1-(5-Phospho-D-ribosyl)-5-amino-4-imidazolecarboxylate->1-(5-Phospho-D-ribosyl)-5-amino-4-imidazolecarboxylate, Carbamoyl phosphate->Carbamoyl phosphate, N->Lactose, N->5-Dehydro-4-deoxy-D-glucarate, 6-Carboxy-5,6,7,8-tetrahydropterin->6-Carboxy-5,6,7,8-tetrahydropterin, N->(9Z,12Z,15Z)-Octadecatrienoic acid, L-Valine->L-Valine, N->2-Acyl-sn-glycero-3-phosphocholine, Menaquinol->Menaquinol, cis-Aconitate->cis-Aconitate, N->Cellulose, Guanine->Guanine, (S)-1-Pyrroline-5-carboxylate->(S)-1-Pyrroline-5-carboxylate, L-Metanephrine->N, 2-Phytyl-1,4-naphthoquinone->2-Phytyl-1,4-naphthoquinone, L-Cysteine->L-Cysteine, 3-Oxopropanoate->3-Oxopropanoate, N->n Maltose, dTDP->dTDP, N->Glycerone, N->Diglucosyldiacylglycerol, N->Glycerophosphoglycoglycerolipid, N->(S)-Methylmalonate semialdehyde, N->1-Organyl-2-lyso-sn-glycero-3-phosphocholine, N->1-Radyl-2-acyl-sn-glycero-3-phosphocholine, N->Carboxylate, N->1-(1-Alkenyl)-sn-glycero-3-phosphoethanolamine, Apoprotein->Apoprotein, N->G13040, N->O-1-Alk-1-enyl-2-acyl-sn-glycero-3-phosphoethanolamine, N->Phosphatidylcholine, N->G09660, N->1-Acyl-sn-glycero-3-phosphocholine, GDP->N, Riboflavin->Riboflavin, L-Histidine->L-Histidine, Molybdopterin->Molybdopterin, Hydroxymethylbilane->Hydroxymethylbilane, alpha-Isopropylmalate->alpha-Isopropylmalate, Pyruvate->Pyruvate, 2-Amino-7,8-dihydro-4-hydroxy-6-(diphosphooxymethyl)pteridine->2-Amino-7,8-dihydro-4-hydroxy-6-(diphosphooxymethyl)pteridine, Orthophosphate->Orthophosphate, Coproporphyrinogen I->Coproporphyrinogen I, (2S,4S)-4-Hydroxy-2,3,4,5-tetrahydrodipicolinate->(2S,4S)-4-Hydroxy-2,3,4,5-tetrahydrodipicolinate, Heme->Heme, L-Allothreonine->L-Allothreonine, N->gamma-L-Glutamylputrescine, N->cis-2-Chloro-4-carboxymethylenebut-2-en-1,4-olide, N->2-Chloromaleylacetate, N->2-Hydroxymuconate, N->gamma-Oxalocrotonate, N->5-Carboxy-2-pentenoyl-CoA, N->(3S)-3-Hydroxyadipyl-CoA, Shikimate->Shikimate, N->L-Arabinose, N->D-Xylulose, 1-(5'-Phosphoribosyl)-5-amino-4-(N-succinocarboxamide)-imidazole->1-(5'-Phosphoribosyl)-5-amino-4-(N-succinocarboxamide)-imidazole, N->D-Xylose, L-Normetanephrine->N, Chloroacetaldehyde->Glycolaldehyde, N->D-Ribulose, N->beta-D-Galactosyl-(1->4)-beta-D-glucosyl-(1<->1)-ceramide, N->D-Galactose, Quinone->Quinone, N->2-Hydroxy-2,4-pentadienoate, N->4-Hydroxy-2-oxopentanoate, dATP->dATP, N->L-Glutamyl 5-phosphate, N->2-Hydroxy-5-methyl-cis,cis-muconate, N->2-Oxo-5-methyl-cis-muconate, 4-Acetamidobutanoate->N-Acetyl-L-glutamate 5-semialdehyde, N->2,5-Dichloro-carboxymethylenebut-2-en-4-olide, Uridine->Uridine, N->2,5-Dichloro-4-oxohex-2-enedioate, 4-Aminobutyraldehyde->4-Aminobutyraldehyde, trans,trans-Farnesyl diphosphate->trans,trans-Farnesyl diphosphate, N->alpha-D-Galactose 1-phosphate, N->beta-D-Glucuronoside, Hydrogen selenide->Hydrogen selenide, N->UTP, Orotidine 5'-phosphate->Orotidine 5'-phosphate, Succinyl-CoA->Succinyl-CoA, Holo-[carboxylase]->Holo-[carboxylase], trans-3-Chloroallyl aldehyde->trans-3-Chloroallyl aldehyde, Nicotinate D-ribonucleotide->Nicotinate D-ribonucleotide, N->D-glycero-beta-D-manno-Heptose 7-phosphate, N->D-Alanyl-D-alanine, 3-Oxo-OPC8-CoA->3-Oxo-OPC8-CoA, N->L-Tyrosine, Geranylgeranyl diphosphate->all-

trans-Polyprenyl diphosphate, N->MurAc(oyl-L-Ala-D-gamma-Glu-L-Lys-D-Ala-D-Ala)-diphospho-undecaprenol, 5-Carboxyamino-1-(5-phospho-D-ribosyl)imidazole->5-Carboxyamino-1-(5-phospho-D-ribosyl)imidazole, Coniferyl alcohol->Coniferyl alcohol, N->Undecaprenyl-diphospho-N-acetylmuramoyl-(N-acetylglucosamine)-L-alanyl-gamma-D-glutamyl-L-lysyl-D-alanyl-D-alanine, N->beta-D-Glucosyl-(1<->1)-ceramide, N->L-Ribulose 5-phosphate, N->D-Xylulose 5-phosphate, N->L-Ribulose, N->Pectate, N->alpha-D-Galactose, beta-D-Glucose 6-phosphate->beta-D-Glucose 6-phosphate, N->ADP-glucose, Porphobilinogen->Porphobilinogen, 3-Methoxy-4-hydroxyphenylglycolaldehyde->Dopamine, LysW-L-glutamate->N-Acetyl-L-glutamate 5-phosphate, N->1,4-beta-D-Xylan, N->Amylose, ATP->ATP, D-Glucosamine 6-phosphate->D-Glucosamine 6-phosphate, Reduced FMN->Reduced FMN, N->Starch, N->Dextrin, N->Maltose, Dihydrofolate->Dihydrofolate, Cob(I)yrinate a,c diamide->Cob(I)yrinate a,c diamide, N->D-Glucuronate, N->Alcohol, beta-D-Fructose 6-phosphate->beta-D-Fructose 6-phosphate, N->D-Fructuronate, 5,10-Methenyltetrahydrofolate->5,10-Methenyltetrahydrofolate, beta-Alanine->beta-Alanine, CTP->N, N-Succinyl-2-L-amino-6-oxoheptanedioate->N-Succinyl-2-L-amino-6-oxoheptanedioate, N->Hexadecanoyl-[acp], N->trans-Hexadec-2-enoyl-[acp], 4-Amino-5-aminomethyl-2-methylpyrimidine->Pyridoxine, (S)-Methylmalonyl-CoA->ADP-L-glycero-beta-D-manno-heptose, Uroporphyrinogen I->Uroporphyrinogen I, 3-Dehydroshikimate->3-Dehydroshikimate, N->D-Galacturonate, N->Pectin, N->n H2O, N->n Methanol, N->D-Altronate, N->D-Tagaturonate, 3-Methoxytyramine->N, N->Protein N(pi)-phospho-L-histidine, N->(S)-3-Hydroxydodecanoyl-CoA, N->Protein histidine, N->(S)-3-Hydroxyoctanoyl-CoA, 2-Phospho-D-glycerate->2-Phospho-D-glycerate, ITP->ITP, N->GDP-4-dehydro-6-deoxy-D-mannose, N->GDP-L-fucose, [Enzyme]-S-sulfanylcysteine->[Enzyme]-S-sulfanylcysteine, N->D-Mannose, N->GDP, Nicotinate->Nicotinate, dUDP->dUDP, 5-Hydroxyindoleacetaldehyde->N, p-Hydroxyphenyl lignin->p-Hydroxyphenyl lignin, Fumarate->Fumarate, OPC4-CoA->OPC4-CoA, Thioredoxin->Thioredoxin, N->(S)-3-Hydroxyhexadecanoyl-CoA, 3-Hydroxyanthranilate->Quinolinate, O-Acetyl-L-homoserine->O-Acetyl-L-homoserine, N->(S)-3-Hydroxytetradecanoyl-CoA, Succinate semialdehyde->Succinate semialdehyde, N->N-Acetyl-D-mannosamine 6-phosphate, N->D-Fructose, N-Acetyl-L-glutamate->N-Acetyl-L-glutamate, Apo-[carboxylase]->Apo-[carboxylase], N->UDP-N-acetyl-3-(1-carboxyvinyl)-D-glucosamine, N->UDP-N-acetylmuramate, Adenylated molybdopterin->Adenylated molybdopterin, (2S)-2-Isopropyl-3-oxosuccinate->(2S)-2-Isopropyl-3-oxosuccinate, myo-Inositol->myo-Inositol, Coproporphyrinogen III->Coproporphyrinogen III, Anthranilate->Anthranilate, Iminoaspartate->Iminoaspartate, H2O->H2O, Glycerone phosphate->Glycerone phosphate, 3-Oxo-OPC6-CoA->3-Oxo-OPC6-CoA, Sarcosine->N, 4-Coumaryl alcohol->4-Coumaryl alcohol, Biotin->Biotin, Phosphatidylglycerol->Phosphatidylglycerol, Formate->Formate, L-Histidinol->L-Histidinol, Propenoyl-CoA->Propenoyl-CoA, Indoleglycerol phosphate->Indoleglycerol phosphate, Sulfur-carrier protein->Sulfur-carrier protein, 3-

(4-Hydroxyphenyl)pyruvate->3-(4-Hydroxyphenyl)pyruvate, Tetrahydrofolate->Tetrahydrofolate, 2-(2-Carboxy-4-methylthiazol-5-yl)ethyl phosphate->2-(2-Carboxy-4-methylthiazol-5-yl)ethyl phosphate, Cytidine->Cytidine, 2,3,4,5-Tetrahydrodipicolinate->2,3,4,5-Tetrahydrodipicolinate, Biotinyl-5'-AMP->Biotinyl-5'-AMP, (R)-3-Hydroxydodecanoyl-[acp]->(R)-3-Hydroxydodecanoyl-[acp], NDP->NDP, D-Glucuronolactone->D-Glucono-1,5-lactone, Hydroxypyruvate->Hydroxypyruvate, Octanoyl-[acp]->N, D-erythro-3-Methylmalate->D-erythro-3-Methylmalate, D-Glucarate->D-Glucarate, L-Glutamyl-tRNA(Gln)->N, 1-(2-Carboxyphenylamino)-1-deoxy-D-ribulose 5-phosphate->1-(2-Carboxyphenylamino)-1-deoxy-D-ribulose 5-phosphate, LysW-L-glutamyl 5-phosphate->4-(L-gamma-Glutamylamino)butanoate, N-Acetylornithine->N-Acetylornithine, CMP->CMP, dUTP->dUTP, (S)-Dihydroorotate->(S)-Dihydroorotate, Crotonoyl-CoA->Crotonoyl-CoA, LysW-gamma-L-alpha-aminoadipate 6-semialdehyde->O-Phospho-4-hydroxy-L-threonine, N-(5-Phospho-D-ribosyl)anthranilate->N-(5-Phospho-D-ribosyl)anthranilate, (2R,3S)-3-Isopropylmalate->(2R,3S)-3-Isopropylmalate, CoA->CoA, Cob(I)alamin->Cob(I)alamin, 2-Phosphoglycolate->2-Phosphoglycolate, D-Glucose 1-phosphate->D-Glucose 1-phosphate, (S)-2-Aceto-2-hydroxybutanoate->(S)-2-Aceto-2-hydroxybutanoate, (R)-Methylmalonyl-CoA->(R)-Methylmalonyl-CoA, alpha-D-Glucose 6-phosphate->alpha-D-Glucose 6-phosphate, 3-Hydroxy-L-kynurenine->(1R,6R)-6-Hydroxy-2-succinylcyclohexa-2,4-diene-1-carboxylate, (+)-7-Isojasmonic acid CoA->(+)-7-Isojasmonic acid CoA, Dephospho-CoA->Dephospho-CoA, 5-Phospho-alpha-D-ribose 1-diphosphate->5-Phospho-alpha-D-ribose 1-diphosphate, N-Acetyl-alpha-D-glucosamine 1-phosphate->N-Acetyl-alpha-D-glucosamine 1-phosphate, Propanoyl-CoA->Propanoyl-CoA, Glyoxylate->N, 5'-S-Methyl-5'-thioinosine->Deoxyinosine, Xanthosine 5'-phosphate->Xanthosine 5'-phosphate, Acceptor->Acceptor, 2-Methylserine->2-Methylserine, 3-Oxododecanoyl-[acp]->3-Oxododecanoyl-[acp], N-Succinyl-LL-2,6-diaminoheptanedioate->N-Succinyl-LL-2,6-diaminoheptanedioate, (R)-2,3-Dihydroxy-3-methylpentanoate->(R)-2,3-Dihydroxy-3-methylpentanoate, 3,4-Dihydroxyphenylethyleneglycol->3,4-Dihydroxyphenylethyleneglycol, Glycolate->Glycolate, AMP->AMP, Cobamide coenzyme->Cobamide coenzyme, GTP->GTP, LysW-L-glutamate 5-semialdehyde->gamma-Glutamyl-gamma-aminobutyraldehyde, Orotate->Orotate, trans-3-Chloro-2-propene-1-ol->trans-3-Chloro-2-propene-1-ol, 5-(2-Hydroxyethyl)-4-methylthiazole->5-(2-Hydroxyethyl)-4-methylthiazole, Uroporphyrinogen III->Uroporphyrinogen III, Deamino-NAD+->Deamino-NAD+, 2-Amino-5-formylamino-6-(5-phospho-D-ribosylamino)pyrimidin-4(3H)-one->Formamidopyrimidine nucleoside triphosphate, N6-(1,2-Dicarboxyethyl)-AMP->N6-(1,2-Dicarboxyethyl)-AMP, dCDP->dCDP, 3,4-Dihydroxymandelaldehyde->3,4-Dihydroxyphenylacetaldehyde, Acetoacetyl-[acp]->Acetoacetyl-[acp], Oxidized flavodoxin->Oxidized flavodoxin, Isopentenyl diphosphate->Isopentenyl diphosphate, 2-Dehydro-3-deoxy-D-gluconate->2-Dehydro-3-deoxy-D-gluconate, Hydrogen peroxide->Hydrogen peroxide, 5'-Deoxyadenosine->Octanoyl-[acp], N->Galactitol, N->Galactitol 1-phosphate, N->D-

Tagatose 6-phosphate, N->D-Tagatose 1,6-bisphosphate, N->2-Dehydro-3-deoxy-6-phospho-D-galactonate, N->Betaine aldehyde, NADPH->NADPH, 3-Ureidopropionate->3-Ureidopropionate, Indole-3-acetaldehyde->Phenylacetaldehyde, 5-O-(1-Carboxyvinyl)-3-phosphoshikimate->5-O-(1-Carboxyvinyl)-3-phosphoshikimate, 2-Polyprenyl-3-methyl-6-methoxy-1,4-benzoquinone->2-Polyprenyl-3-methyl-6-methoxy-1,4-benzoquinone, Syringyl lignin->Syringyl lignin, (3R)-3-Hydroxydecanoyl-[acyl-carrier protein]->(3R)-3-Hydroxydecanoyl-[acyl-carrier protein], trans-Oct-2-enoyl-CoA->trans-Oct-2-enoyl-CoA, Coproporphyrin III->Coproporphyrin III, (S)-3-Methyl-2-oxopentanoic acid->(S)-3-Methyl-2-oxopentanoic acid, Sedoheptulose 1,7-bisphosphate->Sedoheptulose 1,7-bisphosphate, 4-Amino-5-hydroxymethyl-2-methylpyrimidine->4-Amino-5-hydroxymethyl-2-methylpyrimidine, UDP-alpha-D-galactose->UDP-alpha-D-galactose, NAD+->NAD+, L-Ascorbate 6-phosphate->L-Ascorbate 6-phosphate, Phosphatidylethanolamine->Phosphatidylethanolamine, D-Mannose 6-phosphate->D-Mannose 6-phosphate, Reduced electron-transferring flavoprotein->Reduced electron-transferring flavoprotein, Pantetheine 4'-phosphate->Pantetheine 4'-phosphate, Retinol->Retinol, (R)-2-Methylmalate->(R)-2-Methylmalate, L-Homoserine->L-Homoserine, N,N-Dimethylglycine->N,N-Dimethylglycine, 4-Amino-5-hydroxymethyl-2-methylpyrimidine diphosphate->4-Amino-5-hydroxymethyl-2-methylpyrimidine diphosphate, Dolichyl phosphate D-mannose->Lipid X, Inosine->Inosine, 2-Polyprenyl-6-methoxy-1,4-benzoquinone->2-Polyprenyl-6-methoxy-1,4-benzoquinone, Presqualene diphosphate->N, Electron-transferring flavoprotein->Electron-transferring flavoprotein, Dolichyl diphosphate->Linoleate, Indole-3-acetate->Phenylacetic acid, N->3-Oxoadipyl-CoA, N->Iminoglycine, N->4-Fluoromuconolactone, N->2-[(2R,5Z)-2-Carboxy-4-methylthiazol-5(2H)-ylidene]ethyl phosphate, N->Hydrofluoric acid, (3R)-3-Hydroxybutanoyl-[acyl-carrier protein]->(3R)-3-Hydroxybutanoyl-[acyl-carrier protein], N->5-Amino-6-(5'-phosphoribosylamino)uracil, (R)-4-Phosphopantoate->4-Phospho-D-erythronate, 2-Oxoadipate->2-Oxoadipate, Chorismate->Chorismate, 5'-Methylthioadenosine->5'-Methylthioadenosine, Oxygen->Oxygen, Phosphoribosyl-AMP->Phosphoribosyl-AMP, 5,6-Dihydrouracil->5,6-Dihydrouracil, 2-Methylpropanoyl-CoA->(2S,3S)-3-Hydroxy-2-methylbutanoyl-CoA, N->(-)-Ureidoglycolate, N->(S)-4,5-Dihydroxypentane-2,3-dione, N->CO, (S)-3-Hydroxy-3-methylglutaryl-CoA->(S)-Hydroxyhexanoyl-CoA, L-Noradrenaline->N, N->Malonyl-[acp] methyl ester, Retinal->Retinal, Fe-coproporphyrin III->Fe-coproporphyrin III, Reduced ferredoxin->Reduced ferredoxin, N->Enoylglutaryl-[acp] methyl ester, N->Glutaryl-[acp] methyl ester, trans-3-Chloroacrylic acid->Glyoxylate, N->Biotin sulfoxide, N->7-Aminomethyl-7-carbaguanine, Hexanoyl-CoA->Hexanoyl-CoA, N->4-Hydroxybutanoic acid, N->Choline, N->Ascorbate, 1-(5-Phospho-D-ribosyl)-ATP->1-(5-Phospho-D-ribosyl)-ATP, 2-Methylmaleate->2-Methylmaleate, Phosphatidylserine->Phosphatidylserine, L-Aspartate 4-semialdehyde->L-Aspartate 4-semialdehyde, 5-Methyltetrahydropteroyltri-L-glutamate->5-Methyltetrahydropteroyltri-L-glutamate, N->L-Xylulose 5-phosphate, N->Pimeloyl-[acyl-carrier protein], N->Pimeloyl-[acyl-carrier protein] methyl ester, N->Methanol,

L-Isoleucine->L-Isoleucine, Acyl-CoA->Acyl-CoA, N->Enoylpimeloyl-[acp] methyl ester, (R)-4'-Phosphopantothenoyl-L-cysteine->(R)-4'-Phosphopantothenoyl-L-cysteine, L-Glutamyl-tRNA(Glu)->L-Glutamyl-tRNA(Glu), 4-Methyl-5-(2-phosphooxyethyl)thiazole->4-Methyl-5-(2-phosphooxyethyl)thiazole, Betaine->Betaine, 2-Methylprop-2-enoyl-CoA->2-Methylprop-2-enoyl-CoA, dCMP->dCMP, D-Fructose 1,6-bisphosphate->D-Fructose 1,6-bisphosphate, D-Galactonate->D-Galactonate, N->6-Carboxyhexanoyl-CoA, N->Tetradecanoyl-[acp], N->Malonyl-[acyl-carrier protein], N->Acyl-carrier protein, N->Cob(II)alamin, N->trans-Tetradec-2-enoyl-[acp], N->Aquacob(III)alamin, Hydrogen sulfide->Hydrogen sulfide, N->Dodecanoyl-[acyl-carrier protein], N->2,5-Diaminopyrimidine nucleoside triphosphate, N->trans-Dodec-2-enoyl-[acp], 5-Aminolevulinate->5-Aminolevulinate, Malonyl-CoA->Malonyl-CoA, alpha-D-Ribose 1-phosphate->alpha-D-Ribose 1-phosphate, Ethanolamine->Ethanolamine, D-4'-Phosphopantothenate->D-4'-Phosphopantothenate, N->Propanoyl phosphate, LysW-gamma-L-alpha-aminoadipate->N, all-trans-Phytoene->Arachidonate, N->Dethiobiotin, N->Sulfur donor, N->Cellodextrin, N->e-, N->(n-2) H2O, N->(n-2) D-Glucose, 5-(5-Phospho-D-ribosylaminoformimino)-1-(5-phosphoribosyl)-imidazole-4-carboxamide->5-(5-Phospho-D-ribosylaminoformimino)-1-(5-phosphoribosyl)-imidazole-4-carboxamide, N->5'-Deoxyadenosine, N->Cellobiose, N->7,8-Diaminononanoate, N->Maltodextrin, N->8-Amino-7-oxononanoate, N->S-Adenosyl-4-methylthio-2-oxobutanoate, N->L-2-Aminoadipate 6-semialdehyde, Protein N6-(lipoyl)lysine->Protein N6-(lipoyl)lysine, PQQH2->PQQH2, Dopamine->N, 6-Phospho-D-gluconate->6-Phospho-D-gluconate, Sinapyl alcohol->Sinapyl alcohol, (S)-2-Acetolactate->(S)-2-Acetolactate, N->Chitin, N->Chitobiose, N->N-Acetyl-D-glucosamine, Octanoyl-CoA->Octanoyl-CoA, N->Nicotinamide, N->N-Acetyl-D-glucosamine 6-phosphate, N->(R)-Lactate, N->2-Dehydropantoate, N->N-Acetylmuramic acid 6-phosphate, N->Pantothenate, N->gamma-L-Glutamyl-L-2-aminobutyrate, N->Ophthalmate, D-Fructose 6-phosphate->D-Fructose 6-phosphate, 3-Oxohexanoyl-[acp]->3-Oxohexanoyl-[acp], Protein N6-(octanoyl)lysine->N, 4-Phospho-L-aspartate->4-Phospho-L-aspartate, 2-Oxoglutarate->2-Oxoglutarate, Molybdate->Molybdate, LysW-L-ornithine->N2-Succinyl-L-ornithine, Triphosphate->Triphosphate, N->4-Cresol, N->Nicotinamide-beta-riboside, O-Acetyl-L-serine->O-Acetyl-L-serine, N->L-Gulonate, N->Pyridoxal, N->Pyridoxamine, N->N-Succinyl-L-glutamate, Thioredoxin disulfide->Thioredoxin disulfide, N->N-Succinyl-L-glutamate 5-semialdehyde, N->Pyridoxine phosphate, N->Pyridoxal phosphate, N->Pyridoxamine phosphate, (S)-4-Amino-5-oxopentanoate->(S)-4-Amino-5-oxopentanoate, Butanoyl-CoA->Butanoyl-CoA, dADP->dADP, D-Fructose 1-phosphate->D-Fructose 1-phosphate, N->Adenosyl cobinamide, Thiamin monophosphate->Thiamin monophosphate, N->Adenosyl cobinamide phosphate, 6-Pyruvoyltetrahydropterin->6-Pyruvoyltetrahydropterin, N->Sedoheptulose 7-phosphate, N->Adenosine-GDP-cobinamide, N->alpha-Ribazole, N->Sulfate, NADH->NADH, N-Acetyl-D-mannosamine->N-Acetyl-D-mannosamine, N->Adenylyl sulfate, (R)-Mevalonate->N, HCO3-->HCO3-, N->1-Deoxy-D-xylulose 5-phosphate, Shikimate 3-phosphate->Shikimate 3-phosphate, N->CTP, N->Precorrin

2, N->4-(Cytidine 5'-diphospho)-2-C-methyl-D-erythritol, N->2-Phospho-4-(cytidine 5'-diphospho)-2-C-methyl-D-erythritol, N->Sirohydrochlorin, 3-Phosphonooxypyruvate->3-Phosphonooxypyruvate, N->Siroheme, N->2-C-Methyl-D-erythritol 2,4-cyclodiphosphate, N->1-Hydroxy-2-methyl-2-butenyl 4-diphosphate, N->Adenosyl cobyrinate a,c diamide, G00009->2-Deoxy-D-ribose 1-phosphate, N->D-Ribulose 1,5-bisphosphate, OPC6-CoA->OPC6-CoA, Guaiacyl lignin->Guaiacyl lignin, Uracil->Uracil, N->Folinic acid, N->trans-Hex-2-enoyl-[acp], N->Butyryl-[acp], N->But-2-enoyl-[acyl-carrier protein], O-Phospho-L-serine->O-Phospho-L-serine, Molybdoenzyme molybdenum cofactor->Molybdoenzyme molybdenum cofactor, D-Glycerate->D-Glycerate, 3-Oxodecanoyl-CoA->3-Oxodecanoyl-CoA, N->Protoporphyrinogen IX, N->Acetyl-[acyl-carrier protein], (R)-2,3-Dihydroxy-3-methylbutanoate->(R)-2,3-Dihydroxy-3-methylbutanoate, Isocitrate->Isocitrate, 2-Dehydro-3-deoxy-D-galactonate->2-Dehydro-3-deoxy-D-galactonate, N->Bilirubin beta-diglucuronide, N->2,5-Diamino-6-(5'-triphosphoryl-3',4'-trihydroxy-2'-oxopentyl)-amino-4-oxopyrimidine, N->Sucrose, N->2-Amino-4-hydroxy-6-(D-erythro-1,2,3-trihydroxypropyl)-7,8-dihydropteridine, N->Decanoyl-[acp], N->trans-Dec-2-enoyl-[acp], N->trans-Oct-2-enoyl-[acp], (R)-3-Hydroxyhexanoyl-[acp]->(R)-3-Hydroxyhexanoyl-[acp], N->Hexanoyl-[acp], N-Acetyl-L-citrulline->N-Acetyl-L-citrulline]
enzymes matched : total : 537, accurately matched : 425, accuracy : 79.14%
[R00351->R00351, R00352->R00352, R01899->R01899, R00268->R00268, R04779->R04779, R04780->R04780, R09084->R09084, R02073->R02073, R01070->R01070, R01015->R01015, R01061->R01061, R01063->R01063, R01512->R01512, R01518->R01518, R00658->R00658, R00199->R00199, R00200->R00200, R00206->R00206, R02320->R02320, R00209->R00209, R00212->R00212, R01196->R01196, R10866->R10866, R01528->R01528, R10221->R10221, R02736->R02736, R10907->R10907, R02740->R02740, R02739->R02739, R00959->R00959, R00286->R00286, R00291->R00291, R00768->R00768, R05332->R05332, R00420->R00420, R02568->R02568, R01818->R01818, R00885->R00885, R01187->R01187, R07279->R07279, R01334->R01334, R00919->R00919, R04432->R04432, R10161->R10161, R00833->R00833, R00713->R00713, R00714->R00714, R01648->R01648, R10178->R10178, R00093->R00093, R00114->R00114, R00243->R00243, R00248->R00248, R00253->R00253, R00256->R00256, R00483->R00483, R00485->R00485, R00578->R00578, R00220->R00220, R01513->R01513, R00582->R00582, R00480->R00480, R02291->R02291, R01773->R01773, R01775->R01775, R01771->R01771, R01466->R01466, R00751->R00751, R00945->R00945, R04125->R04125, R03425->R03425, R06171->R06171, R00650->R00650, R00946->R00946, R02821->R02821, R04405->R04405, R04858->R04858, R10404->R10404, R00177->R00177, R03105->R03105, R02619->R02619, R00782->R00782, R09776->R00124, R09778->R05085, R00451->R00451, R02735->R02735, R02734->R02734, R04365->R04365, R04198->R04198, R04199->R04199, R10147->R10147, R05052->R05052, R05053->R05053, R04444->R04444, R04445->R04445, R01248->R01248,

| | | | |
|---|---|---|---|
| R01251->R01251, | R01253->R01253, | R00707->R00707, | R00708->R00708, |
| R01398->R01398, | R01086->R01086, | R04035->R04035, | R04037->R04037, |
| R04640->R04640, | R04558->R04558, | R03457->R03457, | R03243->R03243, |
| R01163->R01163, | R03084->R03084, | R02413->R02413, | R02415->R02415, |
| R06847->R06847, | R02412->R02412, | R03460->R03460, | R01714->R01714, |
| R00985->R00985, | R00986->R00986, | R01073->R01073, | R03509->R03509, |
| R03508->R03508, | R01715->R01715, | R01373->R01373, | R00688->R00688, |
| R00689->R00689, | R00692->R00692, | R00694->R00694, | R01728->R01728, |
| R01730->R01730, | R02678->R02536, | R01954->R01954, | R00259->R00259, |
| R00669->R00669, | R02282->R02282, | R01157->R01157, | R00566->R00566, |
| R00996->R00996, | R08648->R08648, | R05070->R05070, | R02197->R02197, |
| R02199->R02199, | R00226->R00226, | R04441->R04441, | R01213->R01213, |
| R04426->R04426, | R01088->R01088, | R01090->R01090, | R01214->R01214, |
| R01215->R01215, | R01434->R01434, | R00927->R00927, | R01278->R01278, |
| R01279->R01279, | R03991->R03991, | R03989->R03989, | R03990->R03990, |
| R03858->R03858, | R03856->R03856, | R03857->R03857, | R04742->R04742, |
| R04753->R04753, | R04754->R04754, | R03778->R03778, | R03776->R03776, |
| R03777->R03777, | R04747->R04747, | R04751->R04751, | R06985->R06985, |
| R01177->R01177, | R01171->R01171, | R01175->R01175, | R01975->R01975, |
| R01976->R01976, | R00238->R00238, | R04543->R04543, | R04566->R04566, |
| R04964->R04964, | R04534->R04534, | R04536->R04536, | R04953->R04953, |
| R04533->R04533, | R01274->R01274, | R01280->R01280, | R01056->R01056, |
| R01829->R01829, | R00762->R00762, | R01068->R01068, | R00214->R00214, |
| R00216->R00216, | R00342->R00342, | R00519->R00519, | R00897->R00897, |
| R01072->R01072, | R04144->R04144, | R04463->R04463, | R04208->R04208, |
| R04591->R04591, | R04559->R04559, | R04560->R04560, | R06975->R06975, |
| R01127->R01127, | R01126->R01126, | R01083->R01083, | R00183->R00183, |
| R00185->R00185, | R00190->R00190, | R00127->R00127, | R02017->R02017, |
| R00124->R02439, | R01137->R01137, | R01138->R01138, | R01130->R01130, |
| R02719->R02719, | R01230->R01230, | R01231->R01231, | R01227->R01227, |
| R01229->R01229, | R01857->R01857, | R01858->R01858, | R00575->R00575, |
| R01397->R01397, | R01993->R01993, | R01867->R01867, | R01868->R01868, |
| R01869->R01869, | R01870->R01870, | R00156->R00124, | R00570->R00124, |
| R00513->R00513, | R00962->R00962, | R01548->R01548, | R02091->R02091, |
| R02096->R02096, | R02371->R02371, | R02372->R02372, | R00964->R00964, |
| R00970->R00970, | R01549->R01549, | R01880->R01880, | R02097->R02097, |
| R02327->R02327, | R02332->R02332, | R01878->R01878, | R01876->R01876, |
| R02269->R02269, | R02325->R02325, | R02100->R02100, | R02094->R02094, |
| R02093->R02093, | R02326->R02326, | R00377->R00377, | R00378->R00378, |
| R00375->R00375, | R00376->R00376, | R00435->R00435, | R00494->R00494, |
| R03599->R03599, | R00907->R00907, | R00908->R00908, | R00965->R00965, |
| R01801->R01801, | R01800->R01800, | R02055->R02055, | R07376->R07376, |
| R07891->R07891, | R07895->R07895, | R07899->R07899, | R01123->R01123, |
| R01658->R01658, | R02003->R02003, | R05233->R05233, | R05234->R05234, |

R00066->R00066, R00549->R00549, R00550->R00550, R00160->R00160, R00161->R00161, R00481->R00481, R07407->R07407, R07410->R07410, R03004->R03004, R03005->R03005, R00189->R00189, R00257->R00257, R01724->R01724, R00104->R00104, R00112->R00112, R04230->R04230, R03269->R03269, R03035->R03035, R03036->R03036, R01074->R01074, R05145->R05145, R00936->R00936, R00939->R00939, R02235->R02235, R02236->R02236, R00937->R00937, R00940->R00940, R03066->R03066, R03067->R03067, R00943->R00943, R04325->R04325, R01655->R01655, R01218->R01218, R01220->R01220, R01225->R01225, R06613->R06613, R02124->R02124, R08379->R08379, R00036->R00036, R00084->R00084, R03165->R03165, R03197->R03197, R00310->R00310, R02272->R02272, R04109->R04109, R05578->R05578, R01717->R01717, R08165->R08165, R04030->R04030, R07263->R07263, R05617->R05617, R06858->R06858, R04993->R04993, R06859->R06859, R04007->R04007, R02596->R02596, R03919->R03919, R01287->R01287, R00895->R00895, R00896->R00896, R00742->R00742, R01786->R01786, R01788->R01788, R02189->R02189, R09085->R09085, R01567->R01567, R01569->R01569, R03055->R03055, R00841->R00841, R00847->R00847, R01186->R01186, R01049->R01049, R00361->R00361, R01082->R01082, R02164->R02164, R00405->R00405, R00727->R00727, R10343->R10343, R00267->R00267, R00709->R00709, R01900->R01900, R01325->R01325, R01547->R01547, R02088->R02088, R02089->R02089, R01967->R01967, R01968->R01968, R04286->R04286, R00966->R00966, R01665->R01665, R01667->R01667, R00441->R00441, R02331->R02331, R02098->R02098, R00710->R00710, R00711->R00711, R00746->R00746, R00754->R00754, R09127->R09127, R01538->R05606, R08572->R08572, R01541->R01541, R03321->R03321, R01324->R01324, R00355->R00355, R00258->R00258, R00396->R00396, R00400->R00400, R02722->R02722, R08774->R08774, R04859->R04859, R07404->R07404, R07405->R07405, R02340->R02340, R00674->R00674, R00229->R00229, R00235->R00235, R02021->R02021, R04457->R04457, R07281->R07281, R05705->R05705, R05706->R05706, R11372->R11372, R03896->R03896, R03898->R03898, R00994->R00994, R03012->R03012, R03033->R03033, R07677->R07677, R10120->R10120, R10116->R10116, R09959->R09959, R09978->R09978, R10002->R10002, R04509->R04509, R03223->R03223, R00615->R00615, R00617->R00617, R00616->R00616, R00618->R00618, R11319->R11319, R03471->R03471, R10712->R10712, R07461->R07461, R00103->R00103, R00137->R00137, R09107->R09107, R01986->R01986, R02549->R02549, R01221->R01221, R10991->R10991, R07390->R07390, R11062->R11062, R11143->R11143, R09395->R09395, R09735->R09735, R11329->R11329, R04972->R04972, R01492->R01492, R07411->R07411]

job time : 8203543ms

total time : 8204180ms
